# Postnatal sustentacular cells as chromaffin progenitors and tumor cells of origin in VHL-related paragangliomas

**DOI:** 10.1038/s41698-025-01145-8

**Published:** 2025-10-15

**Authors:** Petra Bullova, Peng Cui, Maria Arceo, Jiacheng Zhu, Wenyu Li, Monika Plescher, Valentin Poltorachenko, Katerina Stripling, Christian Santangeli, Lidiya Mykhaylechko, Maria Eleni Kastriti, Catharina Larsson, C.Christofer Juhlin, Michael Mints, Susanne Schlisio

**Affiliations:** 1 Department of Oncology-Pathology, Karolinska Institutet, Stockholm, Sweden; 2 Medical University of Vienna, Center for Brain Research, Department of Neuroimmunology, Vienna, Austria; 3 Department of Pathology and Cancer Diagnostics, Karolinska University Hospital, Stockholm, Sweden

**Keywords:** Cancer stem cells, Endocrine cancer, Tumour heterogeneity

## Abstract

The cellular source of chromaffin cell regeneration after birth and its relationship to paraganglioma tumorigenesis remains incompletely defined. Here, we identify a postnatal population of SOX2/SOX10-expressing sustentacular glia-like cells in the organ of Zuckerkandl (OZ) and adrenal gland that give rise to chromaffin cells in vivo. These cells differ transcriptionally from embryonic chromaffin progenitors known as Schwann cell precursors and exhibit a unique progenitor signature. Genetic lineage tracing confirms their postnatal contribution to chromaffin cells, and SOX2^+^PHOX2B^+^ transitional cells were observed in both human and mouse OZ and adrenal tissues. Single-nuclei RNA-seq and inferCNA analysis of pheochromocytoma and paraganglioma (PPGL) revealed that while most sustentacular cells exhibit a stromal profile, a subset in VHL-mutated PPGLs harbor the hallmark 3p chromosomal loss shared with chief tumor cells, suggesting a clonal origin. In an additional PPGL, widespread SOX2 expression in PHOX2B^+^ tumor cells supports this hypothesis. Finally, DLK1-NOTCH signaling was predicted as a central regulator of chromaffin–sustentacular communication, suggesting DLK1 fine-tunes chromaffin regeneration via NOTCH inhibition and may represent a therapeutic target in PPGL.

## Introduction

The adrenal medulla is composed of neural crest-derived neuroendocrine chromaffin cells, which are responsible for catecholamine production, i.e., epinephrine and norepinephrine, mediating the body’s ‘fight or flight’ response. In addition to the adrenal medulla, chromaffin cells are also located in extra-adrenal sites, including the organ of Zuckerkandl (OZ) near the abdominal aorta and in small clusters along paravertebral sympathetic ganglia (paraganglia), producing predominantly norepinephrine^1^. Tumors (PPGL) arising in these tissues, pheochromocytomas (intradrenal) and paragangliomas (extradrenal), often cause catecholamine overproduction, and can lead to severe hypertension and increased risk of cardiovascular events^2^. PPGL have the highest heritability rates among all tumors, with 40% of patients carrying a susceptibility gene mutation in constitutional tissues^3^.

Recent studies have demonstrated that embryonic chromaffin cells at the adrenal anlagen originate from neural crest-derived multipotent Schwann cell precursors (SCPs)^4,5^. SCPs migrate along visceral motor nerves to the developing adrenal anlagen, contributing to the majority of the adrenal chromaffin cell population embryonically^4^. During mouse embryogenesis, SCPs also give rise to paraganglia, including chromaffin cells in the OZ and some sympathetic neurons^6^. Notably, multipotent SCPs persist for a short period during embryonic development^4,5^, disappearing around mouse embryonic day 15, whereas the OZ reaches the largest size just before/after birth^7^ and in humans, this peak occurs even later, around the third year of life before regressing^8^. Thus, the process by which chromaffin cells are generated and replenished postnataly is poorly understood. Putative chromaffin stem cells have been previously postulated^9,10^ and also been recently reported by analyzing deep single-cell sequencing of human postnatal adrenal glands^11,12^. In addition, chromaffin cell division might also repopulate chromaffin cells postnatally^13^.

Here, we describe the existence of a distinct postnatal chromaffin progenitor population using in vivo lineage tracing, single-cell analysis, and immunofluorescence staining. We identified a progenitor population in the mouse adrenal medulla, organ of Zuckerkandl, human adrenal medulla, and PPGL with characteristics of sustentacular glia cells co-expressing SOX10 and SOX2. SOX2 is a well-known transcription factor that maintains multipotency and stemness by regulating genes associated with embryonic neural crest stem cells, embryonic multipotent crest derivatives (SCPs), and postnatal neural stem cells in neurogenic regions of the central nervous system^14,15^. While sustentacular glia cells have traditionally been seen as supportive cells in neuroendocrine tissues through paracrine signaling, our findings suggest they also serve as progenitor cells, contributing to chromaffin cell generation postnatally and supporting neuroendocrine tissue integrity through pathways involving NOTCH inhibitor DLK1 and WNT6 paracrine signaling.

While the majority of sustentacular cells in PPGL are believed to act as non-neoplastic stromal components, we identified in two PPGL cases where a subset of SOX2^+^ sustentacular cells (8% and 15%) shared predicted copy number aberrations with neoplastic tumor cells. This suggests that, in rare instances, sustentacular cells may serve as the tumor cell of origin. These findings not only expand our understanding of postnatal chromaffin progenitors but also highlight the potential dynamic role of sustentacular cells in tumor biology. Investigating their contributions to the tumor microenvironment and progression could inform new diagnostic, prognostic, and therapeutic strategies.

## Results

### Postnatal SOX10^+^ cells are chromaffin progenitor cells in vivo

**Fig. 1:**
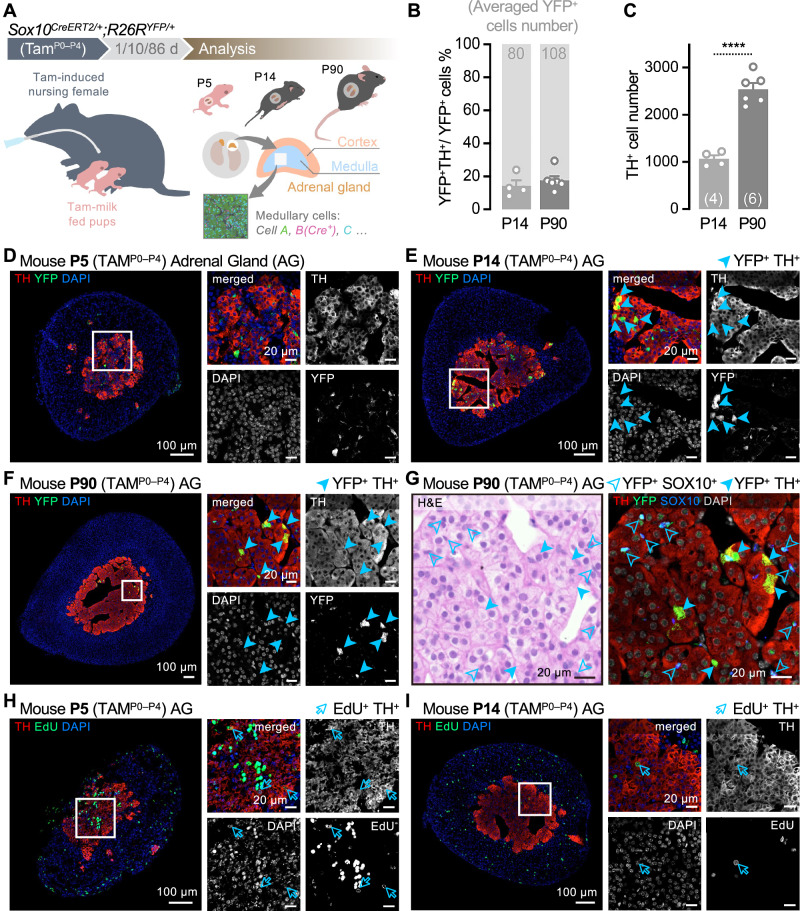
Lineage tracing for SOX10 positive cells in postnatal mouse adrenal glands. **A** Schematic illustrating that tamoxifen (Tam) induced to the nursing female mouse by gavage in the first 4 days (P0–P4) after she gave birth. The *Sox10*^*CreERT2+/−*^*;R26R*^*YFP+/−*^ pups induced with Tam by breast-feeding during these 4 days. After 1, 10, or 86 days, the adrenal glands harvested at P5, P14, or P90 for sectioning, staining, and analysis. **B** Percent stacked bar plot showing the proportion of YFP⁺TH⁺ double-positive cells among total YFP⁺ cells, based on immunofluorescence staining and semi-manual cell counting (*P* value of P14: 14.17% ± 3.40%, *n* = 4; P90: 17.56% ± 2.56%, *n* = 6). Data passed the Shapiro-Wilk normality test (P14: 0.3437; P90: 0.0824), and an unpaired t-test with Welch’s correction revealed no significant difference between P14 and P90 (*P* value = 0.4544). Numbers above each bar indicate the average total number of YFP⁺ cells per time point. Data are presented as mean ± s.e.m. **C** Bar plot showing the total number of medullary TH⁺ cells, based on immunofluorescence staining and semi-manual cell counting (P14: 1067.00 ± 75.14, *n* = 4; P90: 2542.00 ± 128.90, *n* = 6). Normality was confirmed by Shapiro-Wilk test (*P* value of P14: 0.3567; P90: 0.6246), and a t-test with Welch’s correction showed a significant increase in TH⁺ cells at P90 compared to P14 (*P* value < 0.0001). Numbers in each bar indicate sample size (n). Data are presented as mean ± s.e.m. ****, *P* value < 0.0001. **D**–**F** Immunofluorescence staining for the adrenal glands harvested at P5, P14, and P90 after Tam-milk fed. Full arrowheads indicate YFP and TH double-positive (YFP^+^TH^+^) cells. Scale bars in the adrenal gland images and insets are 100 μm and 20 μm, respectively. **G** H&E and immunofluorescent staining for the same window in (**F**). Empty arrowheads indicate YFP and SOX10 double-positive (YFP^+^SOX10^+^) cells showing lineage tracing efficiency. Full arrowheads indicate YFP and TH double-positive (YFP^+^TH^+^) cells. Scale bars are 20 μm. **H**, **I** Immunofluorescent staining for the adrenal glands harvested at P5, and P14 after Tam-milk fed. Empty arrows indicate EdU and TH double-positive (EdU^+^TH^+^) cells. Scale bars in the adrenal gland images and insets are 100 μm and 20 μm. P, postnatal day; Tam, tamoxifen; d, days; TH, tyrosine hydroxylase; YFP, yellow fluorescent protein; DAPI, 4′,6-diamidino-2-phenylindole; EdU, 5-Ethynyl-2’-deoxyuridine; *n*, sample number; *vs*., versus.

To investigate whether SOX10^+^ glial cells contribute postnatally to the generation of chromaffin cells (TH^+^) in the adrenal medulla, we performed fate tracing using the glia-specific inducible Cre-line *Sox10*^*CreERT2+/−*^ coupled to the *R26R*^*YFP+/−*^ reporter. Genetic cell fate tracing was initiated postnatally in glial cells by tamoxifen (TAM)-induced recombination. TAM was administered orally to nursing females via gavage during the first 4 days after birth (P0–P4, Fig. 1A).

Quantification revealed that at postnatal days P14 and P90, 14.17% and 17.56% of YFP⁺ cells, respectively, were positive for tyrosine hydroxylase (TH) (Fig. 1B), with representative immunofluorescence staining shown in Fig. 1D–G. However, the substantial increase in TH^+^ chromaffin cells from P14 and P90 (a 2.5-fold increase, Fig. 1C) could not be attributed entirely to postnatally derived SOX10^+^ cells. This suggests that perhaps neonatal TH^+^ chromaffin cells (P0–P7) retain the capacity to proliferate, contributing to postnatal chromaffin cell expansion. To assess proliferation in the adrenal medulla, we administered EdU (5-ethynyl-2’-deoxyuridine), a thymidine analog that incorporates into newly synthesized DNA, 2 hours before euthanizing P5 and P14 mice. Adrenal glands were sectioned and stained for TH and EdU-labeled cells. At P5, we observed multiple EdU^+^TH^+^ cells in the medullary region (Fig. 1H), indicating that neonatal chromaffin cells retain the capacity for self-renewal. However, the proliferative capacity declined by P14 (Fig. 1I). These findings are consistent with our single-cell RNA-sequencing data, which revealed a proliferative medullary cluster (Cluster 8, Phox2B⁺MKI67⁺) present at E17.5 and P5 but largely absent by P14 (Suppl. Fig. S1B).

Together, these results show that TH⁺ chromaffin cells expand postnatally through both differentiation from SOX10⁺ progenitors and transient self-renewal during the neonatal period.

### Postnatal SOX10^+^ cells in the adrenal medulla and the organ of Zuckerkandl (OZ) co-express the multipotency factor SOX2, resembling sustentacular cells

**Fig. 2:**
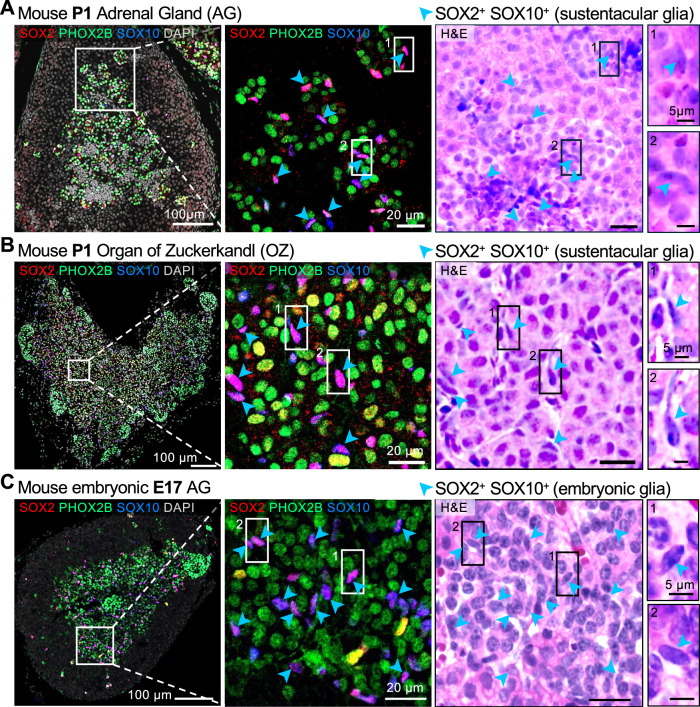
Postnatal SOX2^+^SOX10^+^-expressing cells in the adrenal medulla and organ of Zuckerkandl (OZ) resemble sustentacular glia cells. **A** Immunofluorescence and H&E staining of P1 adrenal gland. Arrows indicate glial cells co-expressing SOX2 and SOX10. Scale bars in the adrenal glands and insets are 100 μm and 20 μm, respectively. **B** Immunofluorescence and H&E staining of P1 Organ of Zuckerkandl (OZ). Arrows indicate glial cells co-expressing SOX2 and SOX10. Scale bars in the Organ of Zuckerkandl and insets are 100 μm and 20 μm, respectively. **C** Immunofluorescence and H&E staining of E17 adrenal gland. Arrows indicate glial cells co-expressing SOX2 and SOX10. Scale bars in the adrenal glands and insets are 100 μm and 20 μm, respectively.

SOX2 co-expression has been observed alongside SOX10 in neural crest cells and embryonic multipotent crest derivatives, SCPs^16–19^. SCPs are the major source for the generation of embryonic chromaffin cells^4^, but not observed postnatally. To characterize chromaffin progenitors, we analyzed both postnatal and embryonic adrenal medulla and OZ for expression of SOX10, SOX2 and sympatho-adrenal lineage specification marker PHOX2B (Fig. 2). Immunofluorescence staining for SOX10 and SOX2 revealed that SOX10^+^ cells are positive for SOX2^+^ in the neonatal (P1) adrenal medulla (Fig. 2A) and OZ (Fig. 2B), persisting to adulthood (Supplementary Fig. S1A), similarly as seen embryonically at day E17 (Fig. 2C). However, hematoxylin and eosin (HE) staining revealed distinctive morphological features of SOX10^+^SOX2^+^ cells in the postnatal medulla and OZ, distinguishing them from the embryonically SOX10^+^SOX2^+^ glia/SCPs cells (E17). Postnatal SOX10^+^SOX2^+^ cells have distinctive spindle-shaped nuclei and extended cellular processes that interface with the surrounding chromaffin cells, and resemble morphological features of sustentacular glia cells (Fig. 2A–B). In contrast, embryonic SOX10^+^SOX2^+^ cells were scattered among differentiating chromaffin cells, having round to oval nuclear shape and cell processes that were thin and difficult to discern (Fig. 2C), indicating that these cells most likely represent satellite-glia and/or SCPs.

**Fig. 3:**
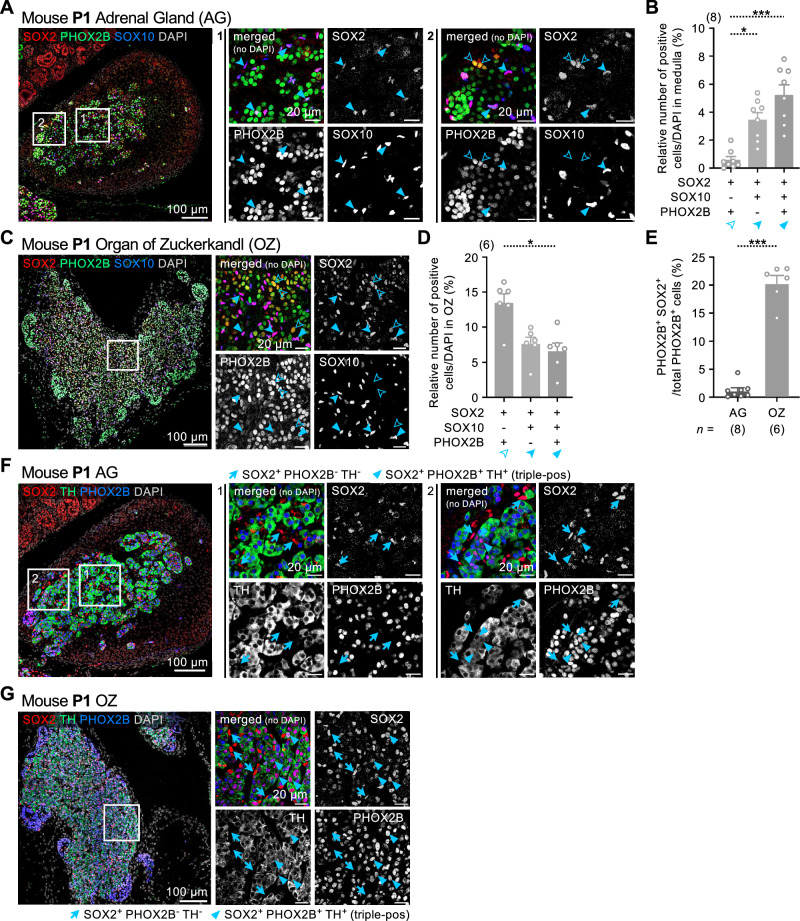
SOX2^+^PHOX2B^+^ double-positive cells in the postnatal adrenal medulla and OZ express tyrosine hydroxylase, indicating a transitional state toward sympatho-adrenal fate. **A** Representative image showing immunofluorescence staining for SOX2, PHOX2B and SOX10 in P1 adrenal gland. Empty arrowheads indicate SOX2^+^PHOX2B^+^ double-positive cells, full arrowheads indicate SOX2^+^SOX10^+^ double-positive glial cells, and full triangles indicate SOX2^+^SOX10^+^PHOX2B^+^ triple-positive glial cells. Scale bars in the adrenal glands and insets are 100 μm and 20 μm, respectively. **B** Percent-stacked bar plot for the ratios of SOX2^+^PHOX2B^+^ double-positive cells (0.6% ± 0.2%), SOX2^+^SOX10^+^ double-positive cells (3.5% ± 0.5%) and SOX2^+^SOX10^+^PHOX2B^+^ triple-positive cells (5.2% ± 0.7%) in total cell number in adrenal medulla. Kruskal-Wallis test; adjusted *P* value from Dunn’s multiple comparisons test for SOX2^+^PHOX2B^+^ vs SOX2^+^SOX10^+^ is 0.0216, and for SOX2^+^PHOX2B^+^ vs SOX2^+^SOX10^+^PHOX2B^+^ is 0.0003; *n* = 8. Data are presented as mean ± s.e.m. *, *P* value < 0.05; ***, *P* value < 0.001. **C** Representative image showing immunofluorescence staining for SOX2, PHOX2B and SOX10 in mouse P1 organ of Zuckerkandl. Empty arrowheads indicate SOX2^+^PHOX2B^+^ double-positive cells, full arrowheads indicate SOX2^+^SOX10^+^ double-positive glial cells, and full triangles indicate SOX2^+^SOX10^+^PHOX2B^+^ triple-positive glial cells. Scale bars in the adrenal glands and insets are 100 μm and 20 μm, respectively. **D** Percent-stacked bar plot for the ratios of SOX2^+^PHOX2B^+^ double-positive cells (13.5% ± 1.3%), SOX2^+^SOX10^+^ double-positive cells (7.6% ± 0.9%) and SOX2^+^SOX10^+^PHOX2B^+^ triple-positive cells (6.5% ± 1.2%) in total cell number in organ of Zuckerkandl. Kruskal–Wallis test; adjusted *P* value from Dunn’s multiple comparisons test for SOX2^+^PHOX2B^+^ vs SOX2^+^SOX10^+^PHOX2B^+^ is 0.0148; *n* = 6. Data are presented as mean ± s.e.m. *, *P* value < 0.05. **E** Percent-stacked bar plot for the ratios of SOX2^+^PHOX2B^+^ double-positive cells in total number of PHOX2B-positive cells in adrenal gland (1.1% ± 0.5%; *n* = 8) vs organ of Zuckerkandl (20.4% ± 1.4%; *n* = 6). Mann-Whitney *t* test; *P* value 0.0007. Data are presented as mean ± s.e.m. ***, *P* value < 0.001. **F** Representative image showing immunofluorescence staining for SOX2, PHOX2B and TH in mouse P1 adrenal gland. Full arrows indicate SOX2 positive cells and full triangles indicate SOX2^+^PHOX2B^+^TH^+^ triple-positive cells. Scale bars in the adrenal glands and insets are 100 μm and 20 μm, respectively. **G** Representative image showing immunofluorescence staining for SOX2, PHOX2B and TH in mouse P1 organ of Zuckerkandl. Full arrows indicate SOX2 positive cells and full triangles indicate SOX2^+^PHOX2B^+^TH^+^ triple-positive cells. Scale bars in the organ of Zuckerkandl and insets are 100 μm and 20 μm, respectively.

Additionally, we observed a few SOX2^+^PHOX2B^+^ cells in the postnatal adrenal medulla and their occurrence was increased in the OZ at P1 (Figs. 2A–B and 3), indicative of a transitional state between progenitors and committed sympatho-adrenal cells. PHOX2B expression indicates lineage commitment to neuroendocrine cell types and is expressed in chromaffin cells, sympathoblast and fully differentiated neurons. However, a SOX2^+^PHOX2B^+^ cell could represent a progenitor cell in transition, a cell that has not yet fully differentiated into chromaffin or sympathetic cells. Thus, we quantified SOX2^+^PHOX2B^+^ double-positive nuclei in the adrenal medulla (Fig. 3A–B) and in the OZ (Fig. 3C–D) at P1. Whereas in the adrenal medulla only a few SOX2^+^PHOX2B^+^ (0.6%) or SOX10^+^SOX2^+^PHOX2B^+^ (5.2%) nuclei were detected (Fig. 3B), a larger proportion of SOX2^+^PHOX2B^+^ double-positive (13.5%) and triple-positive (6.5%) nuclei was observed in the OZ (Fig. 3D–E). Notably, triple-positive cells exhibited lower PHOX2B expression than double-positive SOX2⁺PHOX2B⁺ cells lacking SOX10. This suggests that SOX10 expression may be inversely correlated with PHOX2B levels in this transitional population, possibly reflecting sequential regulation during lineage commitment. This is consistent with previous work demonstrating that SOX10 maintains multipotency and delays overt neuronal differentiation in neural crest stem cells, while still allowing neurogenic transcription factors such as PHOX2B to be induced in a dosage-dependent manner^20^.

We observed a relatively large proportion (20.4%) of PHOX2B positive cells coexpressing SOX2 in the OZ, compared to only 1.1% ± 0.5% in the adrenal gland (Fig. 3E). Notably, SOX2^+^PHOX2B^+^ double-positive cells in both the OZ and adrenal gland expressed TH (triple-positive), consistent with noradrenergic fate restriction (Fig. 3F–G and Supplementary fig. S2A–D).

**Fig. 4:**
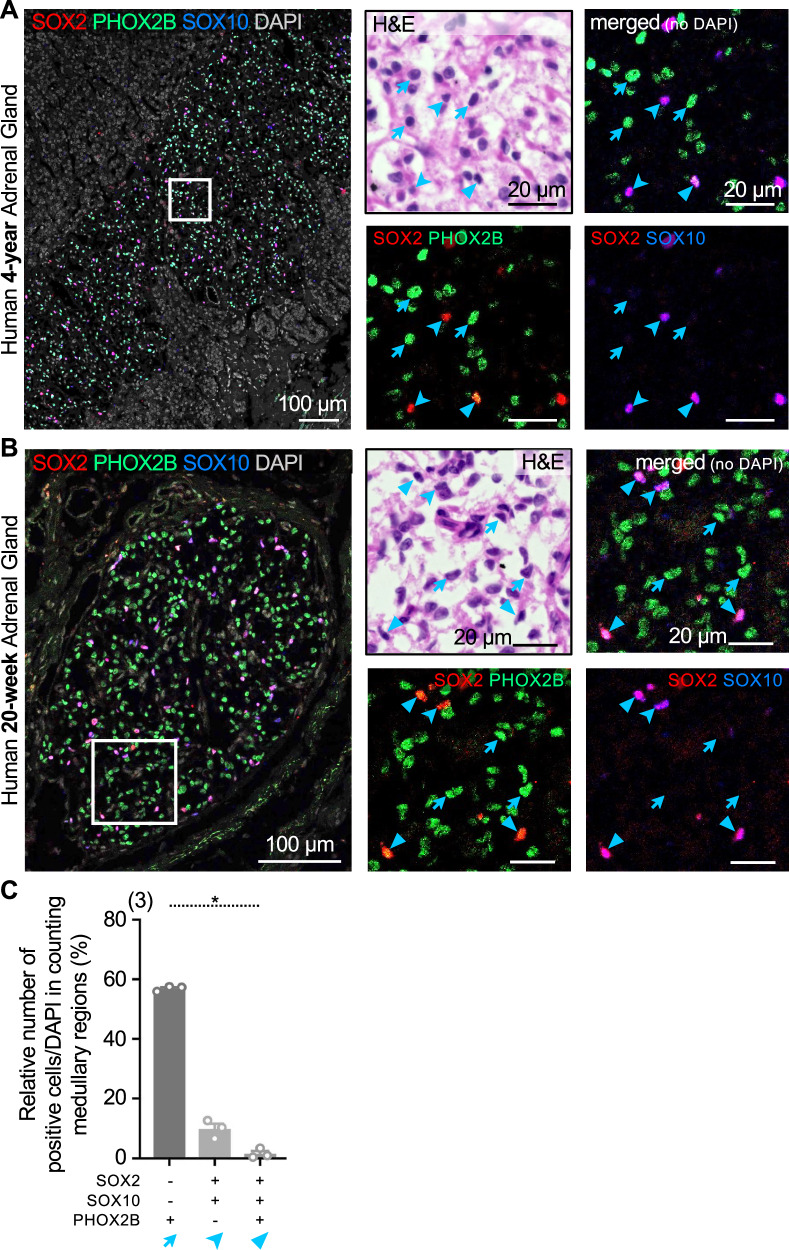
SOX2^+^PHOX2B^+^ transitional cells are present in the human adrenal medulla. **A** Representative image showing H&E and immunofluorescence staining for SOX2, PHOX2B and SOX10 in a 4-year-old human adrenal gland. The box shows one of 5 counting medullary regions in this sample. Full arrows indicate PHOX2B-positive cells, full arrowheads indicate SOX2^+^SOX10^+^ double-positive sustentacular glial cells, and full triangles indicate SOX2^+^SOX10^+^PHOX2B^+^ triple-positive glial cells. Scale bars in the adrenal gland and insets are 100 μm and 20 μm, respectively. **B** Representative image showing H&E and immunofluorescence staining for SOX2, PHOX2B and SOX10 in a 20-week-old human adrenal gland. The box shows one of 5 counting medullary regions in this sample. Full arrows indicate PHOX2B-positive cells, full arrowheads indicate SOX2^+^SOX10^+^ double-positive sustentacular glial cells, and full triangles indicate SOX2^+^SOX10^+^PHOX2B^+^ triple-positive glial cells. Scale bars in the adrenal gland and insets are 100 μm and 20 μm, respectively. **C** Percent-stacked bar plot for the ratios of PHOX2B positive cells (56.89% ± 0.51%, indicated by full arrow), SOX2^+^SOX10^+^ double-positive cells (9.90% ± 1.76%, indicated by full arrowhead) and SOX2^+^SOX10^+^PHOX2B^+^ triple-positive cells (1.60% ± 0.92%, indicated by full triangle) in cell number within all counting medullary regions (3 samples with 5 counting medullary regions each). Kruskal-Wallis test; adjusted *P* value from Dunn’s multiple comparisons test for PHOX2B^+^ *vs* SOX2^+^SOX10^+^ is 0.5391, for PHOX2B^+^ *vs* SOX2^+^SOX10^+^PHOX2B^+^ is 0.0219, and for SOX2^+^SOX10^+^ *vs* SOX2^+^SOX10^+^PHOX2B^+^ is 0.5391; *n* = 3. Data are presented as mean ± s.e.m. *, *P* value < 0.05.

Next, we analyzed adrenal glands from children (*n* = 3), aged 20 weeks to 4 years, to investigate the co-expression of SOX10, SOX2, and PHOX2B and determine whether transitional cells, similar to those observed in mouse adrenal glands, are also present in human tissue (Fig. 4 and Supplementary fig. S3). All SOX10^+^ nuclei co-expressed SOX2, similar to adrenal sustentacular cells in mice (98.22% ± 1.58% of the total SOX10^+^ cells were SOX2^+^). Among all DAPI-positive cells counted, 1.60% ± 0.92% were triple-positive for SOX10, SOX2, and PHOX2B (Fig. 4C), a percentage comparable to that observed in the mouse medulla at P1. These findings suggest that human adrenal chromaffin cells in children may also undergo postnatal regeneration, similar to what is observed in mice. However, the rarity of transitioning cells committing to differentiation aligns with previous findings, which describe the postnatal adrenal medulla as a nearly post-mitotic tissue with a stable and low proliferation rate^13,21^.

### SOX2^+^SOX10^+^ sustentacular cells form a distinct population at postnatal ages with a gene expression program different from embryonic glia/SCPs

**Fig. 5:**
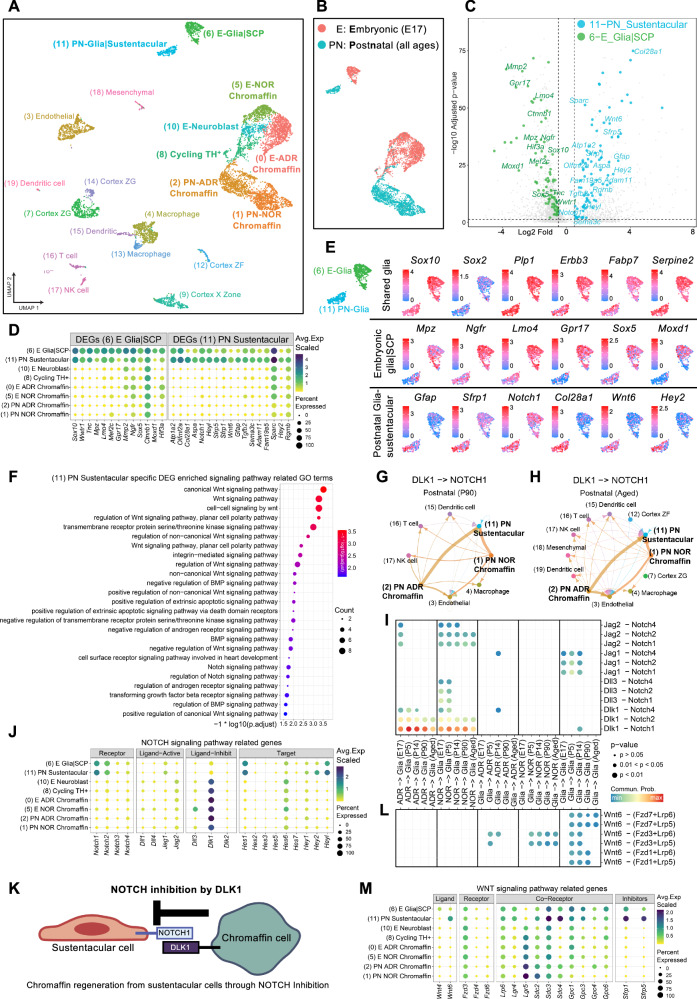
Postnatal sustentacular cells form a distinct population with a gene expression program different from that of embryonic SCPs. **A** UMAP plot showing cellular clusters and their annotations derived from scRNA-Seq data of mouse adrenal tissue. **B** UMAP plot of adrenal medullary cells, color-coded by gross age groups. **C** Volcano plot displaying specific differential expressed genes (DEGs) comparing embryonic (green) and postnatal (blue) glia cells. Genes of interest are labeled with their respective symbols. **D** Dot plot showing the expression of specific DEGs, as labeled in (**C**), in embryonic and postnatal glia cells. **E** UMAP plot illustrating the expression of selected genes representing shared glia markers (upper panel), embryonic glia-specific genes (middle panel), and postnatal glia-specific genes (lower panel). The UMAP plot in the top left corner highlights the two glial cell subsets from (**A**). **F** Bubble plot presenting the top 25 “signaling” related GO terms enriched in the specific DEGs of postnatal glial cells. **G** Network diagram showing inferred DLK1-NOTCH1 ligand-receptor interactions among cell types in P90 mouse cells. **H** Network diagram showing inferred DLK1-NOTCH1 ligand-receptor interactions among cell types in aged mouse cells. **I** Dot plot illustrating ligand-receptor communication pairs related to the NOTCH (upper panel) and WNT (lower panel) signaling pathways between glia cells and chromaffin cells across all five age groups. **J** Dot plot illustrating expression patterns of NOTCH signaling pathway-related genes across identified medullary cell populations. **K** Model for chromaffin cell regeneration from sustentacular cells via NOTCH inhibition. **L** Dot plot illustrating ligand-receptor communication pairs related to the WNT signaling pathways between glia cells and chromaffin cells across all five age groups. **M** Dot plot illustrating expression patterns of WNT signaling pathway-related genes across identified medullary cell populations.

To investigate transcriptional differences between postnatal and embryonic glial cells, we profiled adrenal cell populations in mice across developmental stages. Postnatal (PN) adrenal samples (*n* = 18; ages: P5, P14, P90, and aged) comprised 4552 single cells, while embryonic (E17) medullary samples (*n* = 7) included 2,549 single cells. All cells were analyzed using deep single-cell RNA sequencing with the SmartSeq2 protocol (Fig. 5A, Supplementary fig. S4A–C).

Quality-controlled data were subjected to unsupervised clustering, resulting in the identification of 7,101 adrenal cells and categorized into eight medullary clusters consisting of glia (6,11), chromaffin (0, 1, 2, and 5), embryonic neuroblasts (10) and embryonic TH-expressing cycling cells (8) (Fig. 5A–B, Supplementary fig. S4A–C). We performed in silico subsetting of adrenal medullary cell populations using previously established markers^11^. Clusters were annotated based on the expression of key marker genes; noradrenergic and adrenergic chromaffin markers (*Pnmt, Th, Dbh, Chga, Chgb, Epas1, Penk*), sympathoblast markers (*Elavl4, Isl1, Gap43, Nefl, Prph*), glial markers (*Sox10, Plp1, Erbb3, Fabp7*), and cycling markers (*Top2a, Mki67, Aspm, Bub1*) (Supplementary Fig. S4D). Non-medullary populations, including cortical, mesenchymal, endothelial, and immune cell clusters, were also identified and annotated (Supplementary fig. S4D).

We found that the embryonic glia/SCP population at E17.5 (6-E_Glia/SCP) transcriptionally differed from the postnatal glia/sustentacular population (11-PN_Glia/sustentacular) (Fig. 5C). Differentially expressed genes (DEGs) characterizing these populations were identified using specific DEG analysis methods (log2FC > 0.5, *P.adj* < 0.05, Wilcoxon rank-sum test, Bonferroni correction, Supplementary. Table S1, detailed in Methods), revealing distinct transcriptional programs (Fig. 5C–E and Supplementary Fig. S4E).

The embryonic glial/SCP and postnatal glial sustentacular cells shared the expression of canonical glial markers, including *Sox10, S100b, Foxd3, Plp1, Erbb3, Fabp7* and *Serpine2* (Fig. 5E and Supplementary Fig. S4D). Additionally, they co-expressed *Sox2*, as previously demonstrated by immunofluorescence staining (Fig. 2). However, the embryonic glia/SCP population significantly upregulated *Wwtr1*, *Lmo4*, and *Moxd1* (Fig. 5C–E, Supplementary Fig. S4E and Supplementary Table S1), genes known to play critical roles in maintaining cell stemness and proliferation^22–24^. Notably, *Moxd1* (*Monooxygenase DBH Like 1*) has been previously characterized as highly enriched in embryonic SCPs^25,26^ (Fig. 5E).

In contrast, postnatal glia-sustentacular cells showed significant upregulation of *Wnt6*, *Notch1*, *Hey2*, *Sfrp5/1*, and *Tgfb2* (Fig. 5E, Supplementary Fig. S4E, and Supplementary Table S1). These genes suggest involvement in processes such as cell signaling, fate determination, and maintenance of progenitor-like characteristics^27–33^. Gene ontology (GO) enrichment analysis of the specific DEG list confirmed the enrichment of WNT, BMP, and NOTCH signaling pathways (Fig. 5F and Supplementary Table S2). These findings are consistent with a recent study^12^, which also identified a distinct transcriptional identity for postnatal SOX2^+^ sustentacular cells, differentiating them from embryonic Schwann cell precursors (SCPs). Together, these data emphasize the unique molecular signature of postnatal sustentacular cells and support their role as a specialized glial progenitor population in the adrenal medulla.

### Sustentacular and chromaffin cell-cell communication reveals NOTCH1 and WNT signaling in regeneration and paracrine support

To explore how the identified signaling pathways interact within the adrenal tissue, we performed cell-cell communication analysis using CellChat-tool in different adrenal age groups in mice. CellChat enables systematic analysis of cell-cell communication from single-cell transcriptomics data by quantifying the signaling communication probability between two cell groups, incorporating the core interactions between ligands and receptors^34^.

We observed that the NOTCH signaling pathway was predominantly active in postnatal chromaffin cells (Cluster 1 NOR and Cluster 2 ADR) with receptors mainly on sustentacular cells (Cluster 11), (Supplementary Fig. S4F–G). Analysis of individual ligand-receptor pairs predicted that, across all postnatal ages (P5, P14, P90, and aged), the inhibitory NOTCH ligand DLK1 was primarily expressed and sent by chromaffin cells and received by the Notch1 receptor expressed in sustentacular cells (Fig. 5G–H, Supplementary Fig. S4H). Similarly this communication was also observed in embryonic glia, chromaffin and neuroblast populations at E17 (Supplementary Fig. S4I). DLK1 is a known inhibitor of the NOTCH signaling pathway, acting by preventing activation of the NOTCH receptor^35,36^. Thus, it is plausible that DLK1 expression in postnatal chromaffin cells fine-tunes the regenerative capacity of sustentacular cells during chromaffin differentiation. Notably, DLK1 expression was similarly observed in human neuroendocrine PPGL tumor cells (Supplementary Fig. S5C), suggesting that DLK1 expression in tumor cells might also impact tumor cell differentiation through the tumor microenvironment as observed recently in neuroblastoma^37^.

Furthermore, analysis of all NOTCH ligand-receptor communications in chromaffin and glial populations across developmental stages revealed the expression of NOTCH activating ligand *Jag2* in chromaffin cells (Fig. 5I). NOTCH target genes *Hes1*, *Hey2* and *Heyl* were expressed highly in sustentacular cells (Fig. 5J). This suggests a dual role for NOTCH signaling, involving both inhibitory and activating interactions, which may finetune the regenerative capacity of sustentacular cells (Fig. 5K).

In addition to the identified NOTCH-mediated communication between sustentacular cells and chromaffin cells, WNT signaling emerged as another highly enriched pathway in sustentacular cells, as highlighted by a top-regulated GO term (Fig. 5F). Among the most differentially expressed genes in sustentacular cells were *Wnt6* and *Sfrp1/5* (Fig. 5C–E, Supplementary Fig. S4J). CellChat analysis of WNT signaling pathways predicts ligand-receptor communication between *WNT6* from sustentacular cells, and the receptors *Fzd3/5* and *Lrp5/6* expressed in chromaffin cells during postnatal stages (Fig. 5L and Supplementary Fig. S4K). Interestingly, the expression of *Sfrp1/5* in sustentacular cells suggests a potential dual regulatory role for WNT signaling (Fig. 5M), as these proteins can inhibit WNT receptor activation, balancing inhibitory and activating interactions.

**Fig. 6:**
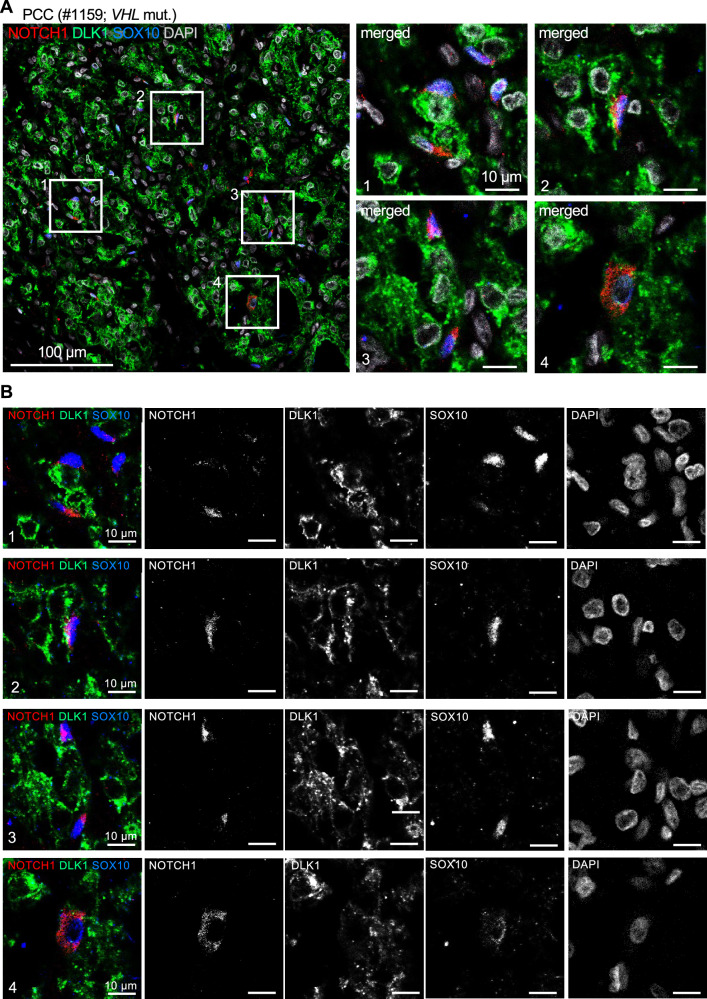
DLK1 and NOTCH1 expression in human PGL reveal cell–cell communication between neuroendocrine tumor cells and sustentacular cells. **A** Immunofluorescence staining for NOTCH1, DLK1, and SOX10 in a human *VHL*-mutated paraganglioma (PCC #1159). Neuroendocrine tumor cells show uniform DLK1 expression (green), while SOX10-expressing sustentacular cells exhibit cytoplasmic localization of NOTCH1 (red). This pattern is consistent with DLK1-mediated inhibition of NOTCH signaling and prevention of NOTCH nuclear translocation. Scale bars: 100 μm (tumor) and 10 μm (insets). **B** Merged (without DAPI) and single marker images of all four ROIs shown in (**A**). Scale bars are 10 μm. ROI, region of interest.

To experimentally validate the predicted DLK1-NOTCH signaling axis, we performed immunofluorescence staining in a human VHL-mutated paraganglioma (sample #1159). Neuroendocrine tumor cells showed uniform DLK1 expression, while SOX10^+^ sustentacular cells exhibited cytoplasmic (non-nuclear) NOTCH1 localization (Fig. 6A–B). This pattern is consistent with DLK1-mediated inhibition of NOTCH signaling, as cytoplasmic retention of NOTCH1 prevents nuclear translocation and downstream transcriptional activation. These findings provide direct validation of our scRNA-seq–based predictions, demonstrating DLK1-NOTCH1–mediated cell–cell communication between neuroendocrine tumor cells and sustentacular cells.

### Deep single-nucleus RNA sequencing analysis of human PPGL shows SOX2 and SOX10 expression in neoplastic cells

**Fig. 7:**
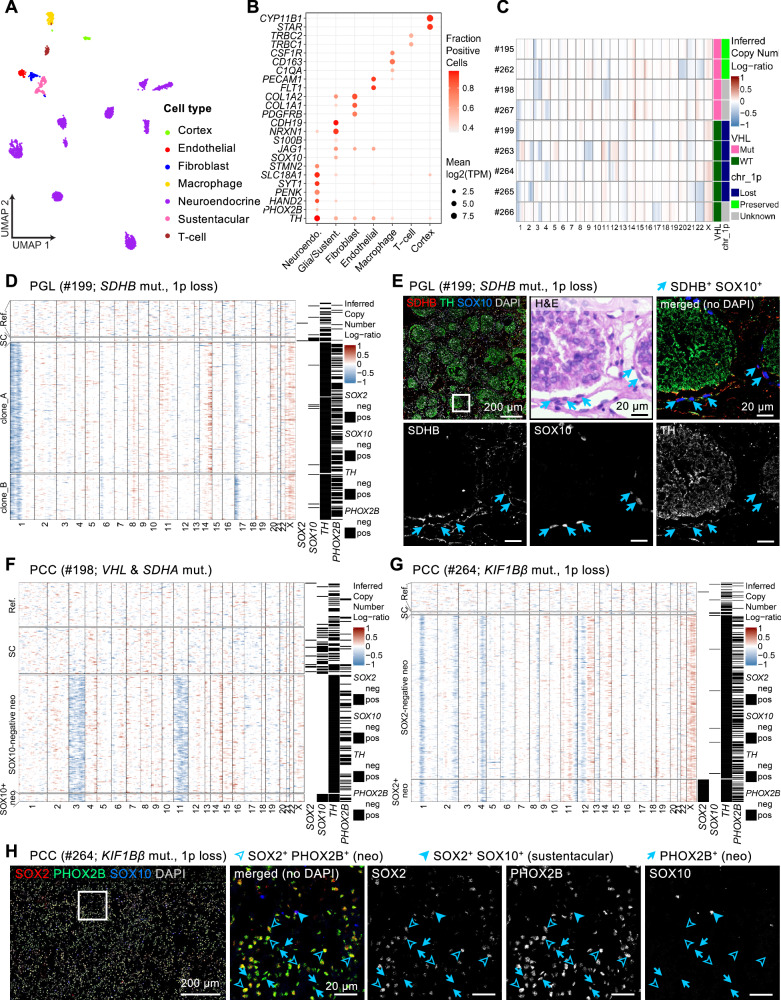
Single-cell RNA sequencing with infer-CNA analysis of human PPGL tumors. **A** UMAP of 2586 single nuclei colored by assigned cell type. **B** Accuracy of cell type assignment. Dot plot showing expression of selected marker genes by cell type. **C** Inferred CNAs match genomic data. For each sample (rows), inferred copy number profiles (red=amplification, blue=deletion) averaged across all malignant cells per sample. Columns are chromosomal positions, averaged into five bins per chromosome. **D** CNA plot of PPGL #199. Each row is a cell, each column a gene, arranged by chromosomal position. Cells are arranged by SOX2-status (SOX2-pos if at least one SOX2 read). Ref, reference; SC, Schwann cells (sustentacular); pos, positive; neg, negative. **E** SDHB, TH and SOX10 immunofluorescence and H&E staining of PPGL #199 as indicated. Arrows indicate sustentacular cells co-expressing SDHB and SOX10, scale bars in the tumor and insets are 200 μm and 20 μm, respectively. **F** CNA plot of PPGL #198. Each row is a cell, each column a gene, arranged by chromosomal position. Neuroendocrine cells are arranged by SOX10 expression (SOX10-pos if at least one SOX10 read). At the top, inferred CNA profiles from all Schwann cells in this sample. **G** CNA plot of PPGL #264. Each row is a cell, each column a gene, arranged by chromosomal position. Cells are arranged by SOX2-status (SOX2-pos if at least one SOX2 read). **H** SOX2, PHOX2B and SOX10 immunofluorescence staining in PPGL #264. Empty arrowheads indicate SOX2^+^PHOX2B^+^ double-positive tumor cells, full arrowheads indicate SOX10^+^SOX2^+^ sustentacular glial cells, and full arrow indicate PHOX2B single positive tumor cells. Scale bars in the tumor and insets are 200 μm and 20 μm, respectively.

Sustentacular cells are commonly observed in PPGL, though their prevalence and characteristics vary depending on the tumor type and its microenvironment. Traditionally considered non-neoplastic glial-like cells, sustentacular cells form a supportive framework around chromaffin cells in both the normal paraganglia and PPGL. However, our genetic tracing revealed that sustentacular glial cells can function as chromaffin progenitor cells postnatally (Fig. 1). To investigate whether they could also serve as the tumor cell of origin, rather than being confined to structural and supportive roles within the tumor microenvironment, we performed deep single-cell sequencing on PPGL (*n* = 9) together with cancer-specific copy number aberration (CNA) inference across both neuroendocrine and sustentacular glial cells. We sequenced 3418 single nuclei from 9 patients and profiled 2586 cells passing quality control (Fig. 7A, Supplementary. Fig. S5A and Methods). After assigning each cell to a cell type (Fig. 7A, B and Supplementary. Fig. S5B, C), we focused on the neuroendocrine compartment to see whether we could find expression of *SOX2* or *SOX10* in neoplastic cells, and the impact of these genes’ expression.

To separate neoplastic from non-neoplastic neuroendocrine cells, copy number aberrations (CNA) were inferred in all neuroendocrine cells, as well as in Schwann cells, the latter to explore whether we can find sustentacular-like neoplastic cells in PPGL. In total 1893/1921 (99%) of all neuroendocrine cells were classified as neoplastic (Supplementary. Fig. S5D), and the inferred patterns of chromosomal aberrations largely confirmed genomic data. All four tumors with genetically confirmed Von Hippel-Lindau (*VHL)* mutations (samples #195, #262, #198 and #267) were found to have a chromosome 3p loss, consistent with a second hit in *VHL*-mutated malignancies^38^ (Fig. 7C). Further validating our approach, the sample #266, with a known *NF1* mutation, showed the corresponding 17q deletion (Fig. 7C and Supplementary Table S3). Similarly, out of the tumors with known chromosome 1p status, inferred copy numbers aligned with known genetics in all cases (Fig. 7C and Supplementary Table S3).

None of the sustentacular cells (SC) sequenced shared any CNA with the neuroendocrine tumor cells and thus were classified as non-neoplastic (Fig. 7D, F–G). In some cases (sample #199 Fig. 7D) only a few sustentacular cells were sequenced. Thus, we additionally performed anti-SDHB immunofluorescence staining on sample #199 (*SDHB* mutant) to validate its neoplastic status (Fig. 7E). PPGL harboring germline *SDHB* mutations are known to display loss of SDHB immunoreactivity^39^ and thus SDHB immuno-staining provides a valuable tool to investigate the neoplastic status of sustentacular cells. Neuroendocrine TH^+^PHOX2B^+^ tumor cells were organized in “zellballen” and confirmed loss of SDHB expression, however surrounding SOX10^+^ sustentacular cells were positive for SDHB, indicating being normal and confirming results derived by inferred CNA (Fig. 7D-E).

While no sustentacular cells were classified as neoplastic, a subset of neoplastic neuroendocrine cells expressed either *SOX10* (sample #198: 6.25%) or *SOX2* (sample #264: 12.1%). Neoplastic cells expressing either *SOX2* (sample #264, Fig. 7G) or *SOX10 (*sample #198 Fig. 7F*)* shared CNA with other cells from the corresponding tumors, and the quantitative metrics used to determine neoplastic-CNA signal and correlation (see Methods)—were comparable across neuroendocrine cells regardless of *SOX2*/*SOX10* positivity (Supplementary. Fig. S5E).

The *SOX10* positive cells were found across multiple tumors, with low numbers in each. In contrast to *SOX10, SOX2* positive neoplastic cells were only found to any significant degree in one tumor, sample #264, where 40/331 (12%) of neoplastic cells had at least one read from *SOX2* (Fig. 7G). Combined immunofluorescence staining for SOX2 and PHOX2B revealed that, in this sample, the majority of neoplastic chief cells (PHOX2B^+^) were SOX2 positive (Fig. 7H and Supplementary. Fig. S5G), indicating that our inability to show *SOX2* mRNA expression in the majority of neuroendocrine cells was an effect of technical dropouts rather than biological heterogeneity within the tumor. Indeed, no genes were significantly differentially expressed between cells with and without identified *SOX2* mRNA in this sample.

To assess the impact of *SOX2* expression in neoplastic cells, we repeatedly performed differential gene expression analysis between this tumor and each of the remaining eight tumors separately, searching for genes that were recurrently over/underexpressed (log2FC > =1, p < 0.05, t-test). 42 genes were significantly overexpressed in sample 264 in all eight pairwise comparisons—notably *ASCL1*, *HOXA9* and *TUBB3*—suggesting a link between malignant *SOX2* expression and a stem-like, neuronal-like phenotype (Fig. S5F, Table S4). Achaete-scute homolog 1 (ASCL1), a proneural transcription factor, plays a central role in neurogenesis^40–42^. In PPGL, sustained ASCL1 expression may promote tumor cell plasticity and maintain an undifferentiated state.

### Schwann cells share 3p deletions with neuroendocrine cells in *VHL*-mutated tumors

Although we could not find any evidence of neoplastic sustentacular cells in our data, the discovery of *SOX2* expression in neuroendocrine neoplastic cells indicated that these cells might exist, but that the low cell numbers sequenced by Smartseq2 and heavy skewing towards neuroendocrine cells in our dataset prevented their discovery. Thus, we re-analyzed a larger, droplet-based single-nucleus RNA sequencing dataset of PPGL samples^43^, providing both more samples and higher numbers of cells per sample.

In five samples with >= 100 Schwann-cell-like cells (SCLCs), as defined by the authors, we re-inferred CNA in SCLCs and neuroendocrine cells, using as reference (Methods) both stromal cells from the same patient as well as, to avoid false positive copy number events due to Schwann-cell-specific transcriptional programs, SCLCs from two normal adrenal medulla samples in the same dataset. SCLCs were classified as malignant if they showed deletions or amplifications consistent with any of the copy number events found in the neuroendocrine cells.

**Fig. 8:**
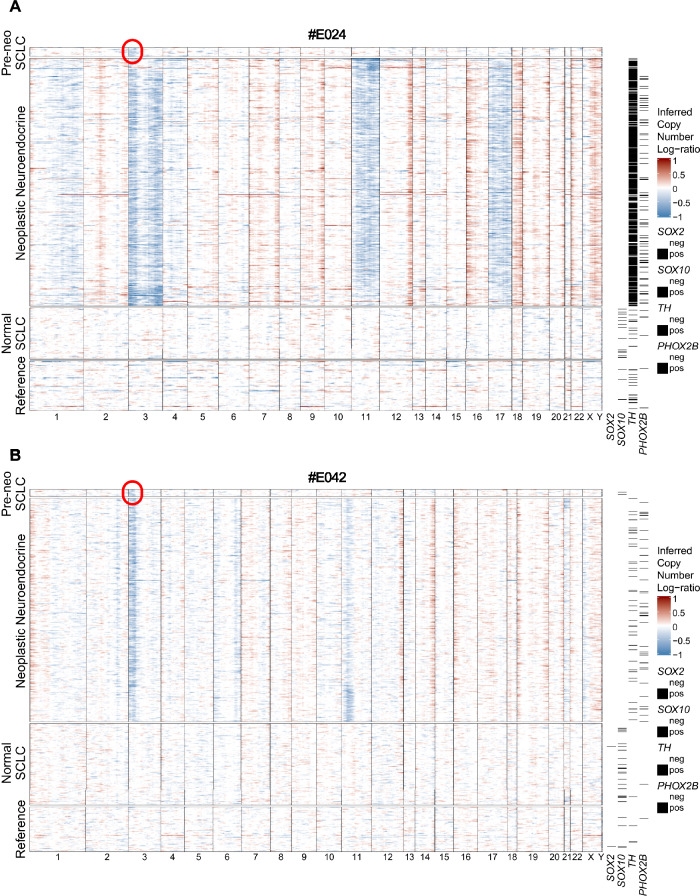
Inferring CNA from VHL-related PPGL reveals pre-neoplastic sustentacular cells sharing 3p deletions. **A**, **B** CNA plot of PPGL #E042 and #E042. Each row is a cell, each column a gene, arranged by chromosomal position. Glia cells (SCLCs) are defined as pre-neoplastic (pre-neo) if they have an inferred 3p deletion (highlighted with a red circle). The reference cells consist of stromal cells from the same patient and SCLCs from normal adrenal medulla. All SCLCs are shown, while 500 neuroendocrine and 100 reference cells were randomly sampled for illustration purposes. Glia cells are refererred to Schwann cell-like cells (SCLCs) from previously defined Zethoven et al.^43^ dataset.

Through this approach, in two samples (#E024, #E042) we were able to find subsets of SCLCs (16/199, 8% and 18/122, 15%) with inferred 3p deletions, consistent with the neuroendocrine cells, but none of the additional copy number events found (Fig. 8A, B). Both samples exhibited germline *VHL* mutations, suggesting that these SCLCs could represent sustentacular glial cells that have undergone a second-hit deletion through the loss of 3p, encompassing the remaining wild-type *VHL* allele. For Von Hippel-Lindau disease-related PPGL, the inactivation or loss of both alleles of the *VHL* gene, as predicted by the Knudson two hit theory, is required. Thus, in both of these cases (#E024, #E042), the biallelic inactivation of *VHL* as an initiating tumor driving event resulted from the combination of germline *VHL* mutation and subsequent chromosomal 3p loss in SCLCs. Analyzing differentially expressed genes between SCLCs with or without 3p deletions, we found that 20 genes were consistently upregulated in non-neoplastic SCLCs in both samples. Notably, only four of these (*CNTN4, BHLHE40*, *RAF1* and *CAPN7*) are located on chromosome 3p, indicating that the inferred 3p deletion is not an artifact of transcriptional downregulation. In “pre-neoplastic” SCLCs, only one gene per sample was significantly upregulated—*HNRNPA2B1* (#E024) and *NRXN1* (#E042) (Supplementary Table S5). Notably, the latter was found to be expressed in both neuroendocrine and Schwann cells in our own PPGL data (Fig. 7B).

## Discussion

Previously, single-nuclei transcriptomes of human adult adrenal glands identified a putative progenitor population with intermediate markers of chromaffin cells, supported by the directional transition from progenitor cells to chromaffin cells predicted by RNA velocity^11^. In this study, we provide in vivo evidence for the existence of such chromaffin progenitor populations postnatally, using in vivo lineage tracing, single-cell RNA sequencing, and immunofluorescence in mouse and human adrenal glands. Our findings establish SOX2^+^SOX10^+^ cells as a distinct progenitor population capable of giving rise to chromaffin cells after birth.

Our single-cell transcriptomic analysis of embryonic and postnatal glial cells revealed that chromaffin regeneration by SOX2^+^SOX10^+^ cells postnatally represents a source distinct from their embryonic origin, the SCPs. While SCPs serve as the primary source of embryonic chromaffin cells during development^4^, their absence postnatally underscores the critical role of an alternative progenitor source in maintaining a growing tissue. Our study provides insights into the role of adrenal SOX2^+^SOX10^+^ glial cells, characterized as sustentacular cells, as postnatal chromaffin progenitors. The identification of SOX2^+^PHOX2B^+^ transitional cells in both human and mouse adrenal medulla, as well as in extra-adrenal chromaffin tissues such as the OZ, further supports the notion of a dynamic and continuous process of chromaffin differentiation during the perinatal and postnatal periods.

The SOX10⁺SOX2⁺ progenitor cells described here differ from the transient “bridge” or “connecting progenitor” states prevouisly reported^4,5^, which represent short-lived intermediates linking SCPs to chromaffin or neuroblast lineages during early development. These transitional states, defined by markers such as *Htr3a*, are absent by mid-gestation in both mouse and human. In contrast, the SOX10⁺SOX2⁺ glial progenitors we identify, including embryonic SCPs and their postnatal derivatives, the sustentacular cells, represent a distinct glial lineage with stable marker expression, indicating a longer-lived progenitor population involved in chromaffin cell maintenance and tumorigenesis.

Single-cell transcriptomic analysis enabled us to quantify the signaling communication of core interactions between ligands and receptors across cell populations. We found that maintaining neuroendocrine tissue integrity involves dynamic signaling interactions of the DLK1-NOTCH and WNT pathways. A recently published study^12^ also describes sustentacular cells as chromaffin stem cells and highlights the role of paracrine signaling through WNT secretion, further supporting our findings.

Furthermore, we characterized the role of SOX2^+^SOX10^+^ sustentacular cells in their contribution to PPGL tumorigenesis, uncovering instances where sustentacular cells may serve as tumor cells of origin. We identified two PPGL samples in which subsets of glial cells (8% and 15%) harbored inferred 3p deletions shared with the neuroendocrine tumor cells, suggesting a common parental cell origin. Both samples harbored germline *VHL* mutations. This supports the notion that a second-hit deletion through the loss of 3p, encompassing the remaining wild-type *VHL* allele, led to *VHL* inactivation—a prerequisite for *VHL* loss-driven tumorigenesis. No additional copy number alterations were shared with the neuroendocrine tumor cells in either case, indicating that 3p loss was an early or initiating event in tumorigenesis, preceding other genetic alterations associated with tumor progression. Thus, we termed these sustentacular cells harboring the 3p deletion as pre-neoplastic. A recent study using CNV predictions also identified rare aneuploid glial cells, termed malignant SCP-like cells, in a related neuroendocrine tumor, neuroblastoma^44^. However, it is possible that this population instead resembles sustentacular glia-like cell, which are also a component of the tumor microenvironment in low-risk neuroblastomas. Nevertheless, given the rarity of PPGL and the limited availability of *VHL*-mutant cases, the prevalence and broader relevance of this finding remain to be determined. Future studies in larger international cohorts will be necessary to determine how frequently 3p loss occurs in sustentacular cells and whether this represents a recurrent initiating event in VHL-driven PPGL.

The reduction or absence of sustentacular cells at metastatic sites, as reported in previous studies, suggests a shift in tumor cell dependency on the microenvironment during disease progression. Our findings support the notion that sustentacular cells help shape the chromaffin tumor niche through paracrine signaling, including DLK1-NOTCH cell–cell interactions and WNT-mediated signaling. Their absence in metastases may reflect a stage at which tumor cells have become independent of these supportive signals. Importantly, while we identified rare sustentacular cells harboring 3p loss and propose these as preneoplastic cells of origin in VHL-mutant PPGL, they lacked additional CNVs present in the tumor bulk and do not fulfill criteria for cancer stem cells.

In one case of PPGL, the majority of neoplastic neuroendocrine PHOX2B^+^ tumor cells co-expressed SOX2. ASCL1, a proneural transcription factor, was found significantly upregulated in this tumor compared to all other samples with SOX2-negative tumor cells. This establishes a link between SOX2 expression and a stem-like phenotype. The regulatory transcription factor Achaete-scute homolog 1 (ASCL1) plays a central role in neurogenesis^40–42,45^. Similarly, ASCL1 might also act as a pioneering factor postnatally, mediating a program in sustentacular progenitor cells that contributes to the chromaffin fate. Notably, the expression of SOX2 in PPGL has previously been reported^46^, supporting a stem-like or progenitor-associated phenotype. Sustained ASCL1 expression in these pheochromocytoma cells may thus reflect an inability of these tumor cells to fully differentiate, keeping them in a plastic and immature state.

In PPGL, the tumor-initiating event is well understood in the context of germline predisposition, but the cell of origin for oncogenic transformation leading to PPGL remains unclear. One of the intriguing aspects of PPGL is their unusually high prevalence of germline predisposition compared to most other adult cancers, despite being a rare cancer type. This has been suggested to be linked to specific developmental windows that permit oncogenic initiation^47^.

While the majority of PPGL are thought to arise from either embryonic or postnatal chromaffin tissue, our findings suggest that tumorigenesis can also be initiated in chromaffin progenitor cells, specifically sustentacular cells. This raises the possibility that developmental timing and cellular plasticity play critical roles in determining susceptibility to oncogenic transformation.

Finally, identifying SOX2^+^SOX10^+^ sustentacular cells as postnatal chromaffin progenitors may have significant clinical implications. Understanding the molecular mechanisms governing their differentiation and plasticity could unveil novel diagnostic and prognostic markers for PPGL, enabling earlier and more precise disease stratification. The presence of SOX2^+^PHOX2B^+^ transitional cells in both adrenal and extra-adrenal chromaffin tissues underscores a dynamic regulatory network, potentially driven by key signaling pathways such as NOTCH inhibition by DLK1 and paracrine WNT signaling, which could serve as therapeutic targets. In this context, the identification of DLK1 expression in chromaffin cells and PPGL is particularly intriguing, as DLK1 has recently been described as an immunotherapeutic target in neuroblastoma, with DLK1 silencing promoting differentiation^37^.

## Methods

### Animals

Mouse experimental procedures were permitted by the Stockholm North committee for animal experiments. *R26R*^*YFP*^ and *Sox10*^*CreERT2*^ mice have been described^48^. C57BL/6 mice were kept in rooms with controlled 12-h light/dark cycles, temperature, and humidity with food and water provided. Animal care were in accordance with the guidelines set by the European Community Council Directives (86/609/EEC).

### Mouse tissue preparation, tamoxifen-induced lineage tracing and EdU administration

P1 animals were anesthetized by i.p. injection of 50 μL pentobarbital sodium (60 mg/mL; APL, #338372) per mouse, and perfused with PBS (Merck, #524650). Kidneys, adrenal glands and organ of Zuckerkandl were dissected out as one compact piece and fixed in 4% paraformaldehyde (PFA; HistoLab Products AB, #02178) at +4°C overnight, with continuous shaking. For postnatal tissue isolation, 5-days old (P5) mice were euthanized by decapitation. Mice older than 13 days, for example the 14-days old (P14) and 90-days old (P90) mice, were euthanized by carbon dioxide inhalation. After euthanasia, bilateral adrenal glands were immediately dissected out. The right adrenal glands were placed in ice-cold 1× PBS, while the left ones were snap-frozen on dry ice. To assess proliferation in adrenal glands, some of the animals were administered with 5-ethyl-2-deoxyuridine (EdU, 2 mg/mL in 1× PBS; Click-iT™ EdU Cell Proliferation Kit for Imaging, Alexa Fluor™ 488 dye, Invitrogen, #C10337) 2 h prior to euthanasia. Adult P90 mice were injected with 400 µg EdU via intraperitoneal (i.p.) injections, while P5 and P14 mice were injected with 100 μg EdU via i.p. injections.

The dissected right adrenal glands were fixed in 4% paraformaldehyde (HistoLab Products AB, #02178) at +4°C for 24 h. After washing with 1× PBS, they were submerged into 10% and 30% sucrose (Merck KGaA, #27480.294) in 1× PBS for cryoprotection for 24–48 h. Afterwards, the adrenal glands were embedded in O.C.T. (Optimal Cutting Temperature; VWR Chemicals, #361603E) and frozen on dry ice. The frozen blocks were stored at –80°C until cryosection was performed. Cryosections of 10 μm were collected on Superfrost PLUS glass tissue slides (Epredia, #J1800AMNZ).

For Tamoxifen-induced lineage tracing, time-mating groups were started in the afternoon. The day of birth was considered as P0. For postnatal induction of *Cre* expression (*Sox10*^*CreERT2*^), oral gavage of the nursing female mouse was performed daily with 2 mg tamoxifen (Sigma-Aldrich, #T5648, dissolved in corn oil [Sigma-Aldrich, #C9267]) for 5 days (P0-4).

### Human tissue specimens and tissue preparation

Tumor tissue samples (PPGL *n* = 9) were collected from individuals operated and diagnosed at the Karolinska University Hospital, Stockholm, Sweden, and previously characterized for mutations in PPGL susceptibility genes^49^. Post-mortem human adrenal glands were obtained from the NIH Neurobiobank (University of Maryland, Baltimore, MD) as previously described^11^. Deep-frozen samples were transferred into ice-cold 4% PFA and fixed at +4°C overnight. The tissue was then processed and embedded in paraffin (Merck, #1.15161), sectioned to 4–5 μm thick sections using HM355S automated microtome (Epredia, #905200) with microtome blades MX35 Ultra (Epredia, #3053835), and dried to Superfrost PLUS glass tissue slides (Epredia, #J1800AMNZ). Alternatively, deep-frozen, fixed samples were embedded in O.C.T. (VWR, #361603E) and sectioned to 10 μm thick sections using Cryotome (CryoStar NX70, Thermo Fisher Scientific).

### Immunofluorescence, RNAscope in situ hybridization, and statistical analyses

Tissue sections were deparaffinized in xylene (HistoLab Products AB, #02070) and rehydrated using a series of ethanol (Solveco) and distilled water washes. Antigen retrieval was performed in citrate target retrieval solution (Agilent, #S1699) for 20 min at 96–100°C. The slides were cooled down, washed, and hydrophobic barrier was created using ImmEdge Pen (Vector laboratories, #H-4000). Alternatively, mouse cryosections were left to dry at room temperature for 15 min and washed 3× in PBS.

The tissues were incubated in blocking buffer with 5% donkey serum (Merck, S30-M) in PBS-T (0.1% Triton X-100; Sigma-Aldrich, #T8787) for 1 h. The tissues were then incubated with primary antibodies diluted in blocking buffer overnight, at +4°C. The antibodies used: chromogranin B (1:500; Atlas Antibodies, #HPA008759), chromogranin B (1:500; Synaptic Systems, #259 103), tyrosine hydroxylase (1:1000; Novus Biologicals, #NB300-110), tyrosine hydroxylase (1:5000; Immunostar, #22914), SOX2 (1:1000; Seven Hills Bioreagents, #WRAB-1236), SOX10 (1:500; R&D Systems, #AF2864), PHOX2B (1:100; R&D Systems, AF4940), PHOX2B (1:50; Santa Cruz, #376997), NEFM (1:300; Abcam, #ab7794) GFP (1:500, Abcam, #ab13970), SDHB (1:200; Proteintech, #67600-1-Ig). After washing the slides 33× in PBS-T, they were incubated with secondary AlexaFluor-conjugated antibodies at room temperature for 1 h in dark. The secondary antibodies used: donkey anti-rabbit AF-555 (#A31572), donkey anti-mouse AF-488 (#A21202), donkey anti-mouse AF-647 (#A31571), donkey anti-goat AF-647 (#A21447), donkey anti-goat AF-488 (#A11055), donkey anti-chicken AF-488 (#A78948), donkey anti-sheep AF-488 (#A11015) donkey anti-sheep AF-555 (#A21436) (all Invitrogen). The tissues were counterstained with 1 μg/mL DAPI (ThermoFisher Scientific, #D3571), incubated with TrueBlack Lipofuscin Autofluorescence Quencher (1:20 in 70% ethanol; Biotium, #23007) for 30 s, and thoroughly washed in PBS. The coverslips were mounted to the slides with ProLong™ Gold Antifade Mountant with DNA Stain DAPI (Invitrogen, #P36935) and allowed to dry.

The images were acquired with ZEISS LSM980-Airy (alternatively, ZEISS LSM800) confocal microscope (Axio Observer Z1/7) and ZEISS ZEN Blue Microscopy acquisition software, v3.2. Quantification was performed semi-manually using QuPath analysis software v0.4.4 (Bankhead et al., 2017) and graphs were created using GraphPad PRISM, v10 for Mac, (GraphPad Software). Statistical analyses were performed using GraphPad Prism version 10.3.1 for macOS (GraphPad Software, La Jolla California USA). For the positive cell co-expression analysis, statistical significance was assessed using the non-parametric Kruskal-Wallis test followed by a post hoc Dunn’s multiple comparison test. For comparison between adrenal glands and organ of Zuckerkandl, non-parametric Mann-Whitney U test was used. For all data sets, the arithmetic mean ± s.e.m. is reported. *P* values < 0.05 were considered statistically significant.

RNAscope in situ hybridization of FFPE tissue was performed according to ACD Bio’s manual using the RNAscope Multiplex Fluorescent Detection Reagent Kit version 2 (#323110). Probes targeting human-specific genes *CHGB* (567681-C1) and *SOX2* (400871-C3) were used and detected with fluorescence dyes Opal 520 (1:1500; Akoya Bioscience, #OP001001) and Opal 690 (1:1500; Akoya Bioscience, #OP001006), respectively.

### Hematoxylin and eosin (H&E) staining

The tissue sections were deparaffinized in xylene (HistoLab Products AB, #02070) and rehydrated using a series of ethanol (Solveco, Sweden) and distilled water washes. When H&E staining was performed on tissues that were previously immunofluorescently labelled, the slides were immersed in PBS and incubated until the cover slips detached. Afterwards, they were incubated in hematoxylin (HistoLab Products AB, #01820) for 5 min followed by a thorough wash in tap water. The slides were then incubated in eosin (HistoLab Products AB, #01650) for 45 s, dehydrated in 100% ethanol and xylene, and coverslips were mounted with VectaMount Mounting Medium (Vector Laboratories, #H-5000). The images were acquired with ZEISS Axio Scan.Z1 Slide Scanner and ZEISS ZEN Blue Microscopy acquisition software, v3.1.

### Mouse single-cell RNA sequencing, cell isolation, flow cytometry and quality control

For single-cell suspension preparation and YFP^+^ cells FACS sorting, embryonic adrenal glands (E17.5) were dissected from *Sox10*^*CreERT2+/−*^*;R26R*^*YFP+/−*^ embryos and placed in 1 mL cold LBSS buffer (154 mM NaCl, 5 mM KCl, 3.6 mM NaHCO₃, 5 mM HEPES, 11 mM glucose) on ice. After dissecting the adrenal glands open to expose the medulla, tissues were washed in LBSS (3×) and incubated in 500 μL of pre-warmed papain solution (25 U/mL in dissociation medium: DMEM/F12 without phenol red, supplemented with HEPES, 1mM L-cysteine, 1 mM CaCl₂, and 1 mM EDTA) for 30 minutes at 37°C.

Following enzymatic digestion, tissues were washed three times in trituration buffer (DMEM/F12 without phenol red, with HEPES, 2% horse serum, 1 mM CaCl₂, 1 mM EDTA), and then resuspended in 500 μL of trituration medium supplemented with 12.5 μL RNasin® Plus Ribonuclease Inhibitor (Promega, N2615). Cells were gently triturated using three fire-polished Pasteur pipettes of decreasing diameter and filtered through a 40 μm strainer, resuspended in FACS buffer (PBS with 2% FBS and 1 mM EDTA) and stained with propidium iodide to exclude dead cells. Initial gating on forward scatter area (FSC-A) vs. side scatter area (SSC-A) was performed to exclude cellular debris and non-viable cells. Subsequent gating on forward scatter area (FSC-A) vs. forward scatter width (FSC-W) was used for singlet discrimination to eliminate cell aggregates. YFP⁺ cells were isolated based on their fluorescence signal, representing the traced Sox10-lineage cell population.

Viable YFP⁺ single cells were sorted into 384-well plates pre-filled with Smart-seq2 lysis buffer. Plates were sealed, immediately placed on dry ice, and sent to the Eukaryotic Single Cell Genomics (ESCG) facility at SciLifeLab for downstream processing and library preparation using the Smart-seq2 protocol. Single-cell raw sequencing data was pre-processed as described in our previous work^11^. The quality control of mice individual cells included removing cells expressing more than 10,000 or fewer than 200 genes, removing cells whose total expression was due to mitochondrial genes in more than 10%, and removing from the expression matrix genes that are expressed in fewer than 3 cells. Mouse single-cell data clustering and cell type identification are further described in supplementary materials.

### Mouse single-cell data clustering and cell type identification

Gene expression of cells was log normalized, standardized and scaled using Seurat v4.4.0 in R v4.3.1 with NormalizeData() and ScaleData(). Cell cycle score was calculated for each and all cells using G2M and S gene sets provided with the package and as previously described^50^. We adjusted gene expression by regressing out the effect of cell cycle, mitochondrial expression, and library size. We identified highly variable genes with FindVariableFeatures() using nfeatures=2500. We then clustered the data with FindClusters() using Resolution=0.6. The mutual nearest neighbors were calculated using FindNeighbors() with dims=40 principal components and k.param=20 neighbors. Unless otherwise specified parameters used were default. We then identified *Sox2*^+^ cells in glial clusters 6 (corresponding to E17 cells) and 11 (corresponding to P5, P14, P90, and aged cells) as cells showing standardized gene expression larger than 0.

Cell clusters identified in the single-cell dataset were annotated based on the known cell type marker genes^11^.

### Specific differential expressed genes (DEGs) and GO term pathway analysis

To identify specific DEGs in mouse embryonic and postnatal glia cells, we conducted differential expression analysis by comparing each cellular cluster against another cluster in a pairwise manner. We used the “FindMarkers()” function from the Seurat R package^51^ for this analysis, employing the default Wilcoxon rank-sum test. *P* values were adjusted for multiple testing using the Bonferroni correction, and genes with adjusted *P* values below 0.05 and log fold change above 0.5 as significant DEGs. To identify specific DEGs, we took the intersection of upregulated DEG lists from all pairwise comparisons involving a given cluster. Genes present in the final intersections were classified as specific DEGs for that cluster.

Gene-ontology enrichment analysis was performed using the ClusterProfiler R package^52^. Specifically, we applied the “enrichGO()” function using the selected gene list as inputs and performing enrichment analysis based on the hypergeometric test. Multiple testing correction was conducted using the Benjamini-Hochberg (BH) method with significant enriched GO terms selected based on adjusted *P* values below 0.05. We used ‘org.Mm.eg.db’ database for genome-wide annotation for the mouse genome.

### Cellular interaction network

We inferred cell-cell-communication network using the CellChat R package^53^ for each age in this study. Specifically, we performed two parallel analysis using wild-type mouse data. (a) Global Cell Type Interactions: Cell types comprising at least 10 cells in each age group were analyzed (Figs. 5G and H, Supplementary. Fig. S5E, G, H, J). (b) Subgroup-Specific Interactions: We focused on ADR chromaffin cells, NOR chromaffin cells, and glial cells in each age group (Fig. 5I).

### Human single-nuclei sequencing, pre-processing and quality control

Human samples were processed using a single-nuclei sequencing protocol, while mouse samples underwent a single-cell sequencing protocol. The protocols for processing both human and mouse samples were described in our previous work^11^.

Human single-nuclei raw sequencing data were hard-clipped to remove adapters and reads shorter than 20 bases with TrimGalore v0.6.1. FastQC v0.11.8 (https://www.bioinformatics.babraham.ac.uk/projects/fastqc) was used to further remove low quality reads, such that reads failing 3 or more of the tests were excluded. After quality control, the remaining high-quality reads were aligned to the human transcriptome (hg38, GRCh38.p13) with STAR v2.7.11a. Transcripts were counted and gene length-normalized to transcripts per million (TPM) with RSEM v1.1.3^54^ with standard parameters in single-cell mode (--single-cell-prior). Cells underwent quality control by using cutoffs of at least 1000 genes/cell and a mitochondrial fraction <30%, 523 ( ~ 15%) low-quality cells were filtered out. For all downstream analyses, TPM matrices were taken from only the cells relevant to the analysis, log2((TPM/10) + 1)-transformed as in ref. ^55^, and only genes with an average expression of >4.5 log2(TPM) across all cells in the matrix were kept. Thereafter, gene expression levels per cell were mean-centered by the average expression of each gene.

Human single-nuclei data clustering and cell type assignment were performed as followed: A matrix of 7464 genes x 2895 cells was created as per above from all cells passing initial QC. Thereafter, the matrix underwent UMAP dimensionality reduction and DBscan clustering (minpts=10). Each cluster was roughly assigned an initial cluster type based on the top 50 differentially expressed genes in that cluster compared to all other cells (Supplementary. Table S6).

Since our dataset was heavily enriched in neuroendocrine cells, impacting the clustering of other cell types, all non-neuroendocrine cells were then separately clustered to accurately assign microenvironment cell type (Supplementary. Table S7). Upon this second clustering, 111 cells were removed due to forming patient-specific clusters with mixed neuroendocrine-immune signatures, indicating doublet formation. FACS analysis also confirmed that these samples were enriched in S phase—increasing the risk of doublet formation due to uptake of PI stain. Finally, performing CNA analysis on these cells showed weaker CNA patterns plus a 6p amplification, typical of immune cells due to the HLA locus, in cells assigned as doublets (data not shown). Additionally, a group of 198 TME cells, having few differentially expressed genes and significantly enriched in noncoding genes to other cells (35% vs 20%, *P* value < 2.2E-16, t-test), was removed as low-quality cells.

### Copy number aberration (CNA) inference

Copy number aberration (CNA) inference was performed similarly to^56^. Since not all samples had enough stromal cells to be used as reference for CNA inference, a pooled reference of fibroblasts, endothelial cells adrenocortical cells, deriving from multiple patients was created. To avoid creating batch effects, from each patient with at least 15 fibroblasts, endothelial or adrenocortical cells, 15 cells from that cell type were randomly sampled. This created a pooled reference comprising 30 fibroblasts, 45 endothelial cells and 30 adrenocortical cells, which was used for all CNA inference.

For each patient, this reference was added to all neuroendocrine and Schwann cells from that patient to create a new matrix, which was filtered and centered as earlier described. For reanalysis of the Zethoven et al.^55^, dataset, adrenocortical, chromaffin, endothelial cells and fibroblasts from the same patient, as well as all SCLCs from two normal adrenal medulla samples were instead used as reference. Genes were reordered by chromosomal location and normalized expression values were limited to −3 and 3. For each cell, a moving 100-gene average was calculated at each chromosomal position, giving initial CNA values.

For each of the three reference cell types, a mean CNA value was taken, averaging the moving averages across all cells from that type. To infer relative copy number changes compared to cells with a normal karyotype, the highest of these three values was then subtracted from all positive CNA values and the lowest was subtracted from all negative values. Additionally, all values between −0.15 and 0.15 were assumed to reflect noise and set to zero.

To infer genetic subclones, the top 2/3 of the CNA matrix by absolute value of the CNA signal underwent UMAP dimension reduction and overclustering using graph-based Louvain clustering (k = 15). An average CNA value per cluster per chromosome arm was then calculated and deletion/amplification was defined by average CNA value < −0.15 or >0.15, respectively. Clusters were then merged if their deletion/amplification events were the same across all chromosome arms. This was repeated, and new average CNA values calculated, until the remaining clusters differed by at least one chromosome arm.

### Neoplastic cell definitions

To stringently define neoplastic cells, we used CNA signal and CNA correlation as quantitative metrics. CNA signal was defined as mean of CNA absolute values across the top 20% genes by CNA absolute value. Then, for each cell, a correlation coefficient, called CNA correlation, was calculated between the CNA values of that cell and the average CNA profile of the top 20% cells by CNA signal. Cutoffs for each metric were set at median +2 SD of the reference cells, and only cells passing both cutoffs were classified as malignant (Supplementary. Fig. S5B).

### Ethics approval

Collection and analyses of human samples (PPGL) are covered by the ethical approval numbers 01-136 local ethical committee KI forskningsetikkommitté Nord) and 2020-04226 (Swedish Ethical Review Authority). Post-mortem human adrenal glands for staining’s were obtained from the NIH Neurobiobank (University of Maryland, MD) under the same ethical permit from Stockholm Regional Ethical Review Board and the Karolinska University Hospital Research Ethics Committee (KI 2007/069 and KI 2001/136). Ethical permits for animal studies were approved by the appropriate local and national authorities (Jordbruksverket, Sweden).

## Supplementary information

Supplementary Information

Supplementary data 1

## Data Availability

Raw single-cell/nuclei sequencing data have been submitted to the European Genome-phenome Archive (EGA) Database with accession number EGAD50000001689. The analysis pipeline has been uploaded to GitHub and is publicly available at: https://github.com/susanneschlisio/Bullova-et-al. The raw sequencing data of human and mouse samples will be publicly accessible on the date of publication.

## References

[1] Kastriti, M. E., Kameneva, P. & Adameyko, I. Stem cells, evolutionary aspects and pathology of the adrenal medulla: a new developmental paradigm. *Mol. Cell Endocrinol.* **518**, 110998 (2020). 32818585 10.1016/j.mce.2020.110998

[2] Lenders, J. W., Eisenhofer, G., Mannelli, M. & Pacak, K. Phaeochromocytoma. *Lancet* **366**, 665–675 (2005). 16112304 10.1016/S0140-6736(05)67139-5

[3] Crona, J., Taieb, D. & Pacak, K. New perspectives on pheochromocytoma and paraganglioma: toward a molecular classification. *Endocr. Rev.* **38**, 489–515 (2017). 28938417 10.1210/er.2017-00062 PMC5716829

[4] Furlan, A. et al. Multipotent peripheral glial cells generate neuroendocrine cells of the adrenal medulla. *Science* **357**, eaal3753 (2017). 28684471 10.1126/science.aal3753 PMC6013038

[5] Jansky, S. et al. Single-cell transcriptomic analyses provide insights into the developmental origins of neuroblastoma. *Nat. Genet* **53**, 683–693 (2021). 33767450 10.1038/s41588-021-00806-1

[6] Kastriti, M. E. et al. Schwann cell precursors generate the majority of chromaffin cells in Zuckerkandl organ and some sympathetic neurons in paraganglia. *Front. Mol. Neurosci.* **12**, 6 (2019). 30740044 10.3389/fnmol.2019.00006 PMC6355685

[7] Schober, A. et al. Cell loss and autophagy in the extra-adrenal chromaffin organ of Zuckerkandl are regulated by glucocorticoid signalling. *J. Neuroendocrinol.* **25**, 34–47 (2013). 23078542 10.1111/j.1365-2826.2012.02367.x PMC3564403

[8] Salgaonkar, H. et al. Laparoscopic resection of a large paraganglioma arising in the organ of Zuckerkandl: report of a case and review of the literature. *J. Minim. Access Surg.* **12**, 378–381 (2016). 27251804 10.4103/0972-9941.169990 PMC5022524

[9] Chung, K. F. et al. Isolation of neural crest derived chromaffin progenitors from adult adrenal medulla. *Stem Cells* **27**, 2602–2613 (2009). 19609938 10.1002/stem.180

[10] Rubin de Celis, M. F. et al. Multipotent glia-like stem cells mediate stress adaptation. *Stem Cells* **33**, 2037–2051 (2015). 25802118 10.1002/stem.2002

[11] Bedoya-Reina, O. C. et al. Single-nuclei transcriptomes from human adrenal gland reveal distinct cellular identities of low and high-risk neuroblastoma tumors. *Nat. Commun.* **12**, 5309 (2021). 34493726 10.1038/s41467-021-24870-7 PMC8423786

[12] Santambrogio, A. et al. SOX2(+) sustentacular cells are stem cells of the postnatal adrenal medulla. *Nat. Commun.* **16**, 16 (2025). 39747853 10.1038/s41467-024-55289-5 PMC11696870

[13] Tischler, A. S., Ruzicka, L. A., Donahue, S. R. & DeLellis, R. A. Chromaffin cell proliferation in the adult rat adrenal medulla. *Int. J. Dev. Neurosci.* **7**, 439–448 (1989). 2816483 10.1016/0736-5748(89)90004-x

[14] Graham, V., Khudyakov, J., Ellis, P. & Pevny, L. SOX2 functions to maintain neural progenitor identity. *Neuron* **39**, 749–765 (2003). 12948443 10.1016/s0896-6273(03)00497-5

[15] Arnold, K. et al. Sox2(+) adult stem and progenitor cells are important for tissue regeneration and survival of mice. *Cell Stem Cell* **9**, 317–329 (2011). 21982232 10.1016/j.stem.2011.09.001 PMC3538360

[16] Le, N. et al. Analysis of congenital hypomyelinating Egr2Lo/Lo nerves identifies Sox2 as an inhibitor of Schwann cell differentiation and myelination. *Proc. Natl. Acad. Sci. USA* **102**, 2596–2601 (2005). 15695336 10.1073/pnas.0407836102 PMC548989

[17] Pevny, L. & Placzek, M. SOX genes and neural progenitor identity. *Curr. Opin. Neurobiol.* **15**, 7–13 (2005). 15721738 10.1016/j.conb.2005.01.016

[18] Sarkar, A. & Hochedlinger, K. The sox family of transcription factors: versatile regulators of stem and progenitor cell fate. *Cell Stem Cell* **12**, 15–30 (2013). 23290134 10.1016/j.stem.2012.12.007 PMC3608206

[19] Wakamatsu, Y., Endo, Y., Osumi, N. & Weston, J. A. Multiple roles of Sox2, an HMG-box transcription factor in avian neural crest development. *Dev. Dyn.* **229**, 74–86 (2004). 14699579 10.1002/dvdy.10498

[20] Kim, J., Lo, L., Dormand, E. & Anderson, D. J. SOX10 maintains multipotency and inhibits neuronal differentiation of neural crest stem cells. *Neuron* **38**, 17–31 (2003). 12691661 10.1016/s0896-6273(03)00163-6

[21] Tischler, A. S., DeLellis, R. A., Nunnemacher, G. & Wolfe, H. J. Acute stimulation of chromaffin cell proliferation in the adult rat adrenal medulla. *Lab. Invest.* **58**, 733–735 (1988). 3379918

[22] Shi, P. et al. MOXD1 knockdown suppresses the proliferation and tumor growth of glioblastoma cells via ER stress-inducing apoptosis. *Cell Death Discov.* **8**, 174 (2022). 35393406 10.1038/s41420-022-00976-9 PMC8991257

[23] Ochoa, S. D., Salvador, S. & LaBonne, C. The LIM adaptor protein LMO4 is an essential regulator of neural crest development. *Dev. Biol.* **361**, 313–325 (2012). 22119055 10.1016/j.ydbio.2011.10.034 PMC3738297

[24] Chen, Y. A. et al. WW domain-containing proteins YAP and TAZ in the hippo pathway as key regulators in stemness maintenance, tissue homeostasis, and tumorigenesis. *Front. Oncol.* **9**, 60 (2019). 30805310 10.3389/fonc.2019.00060 PMC6378284

[25] Kastriti, M. E. et al. Schwann cell precursors represent a neural crest-like state with biased multipotency. *EMBO J.* **41**, e108780 (2022). 35815410 10.15252/embj.2021108780 PMC9434083

[26] Fredlund, E. et al. MOXD1 is a lineage-specific gene and a tumor suppressor in neuroblastoma. *Sci. Adv.* **10**, eado1583 (2024). 38905335 10.1126/sciadv.ado1583 PMC11192077

[27] Wei, M. et al. Expression and function of WNT6: from development to disease. *Front. Cell Dev. Biol.* **8**, 558155 (2020). 33425886 10.3389/fcell.2020.558155 PMC7794017

[28] Bovolenta, P., Esteve, P., Ruiz, J. M., Cisneros, E. & Lopez-Rios, J. Beyond Wnt inhibition: new functions of secreted Frizzled-related proteins in development and disease. *J. Cell Sci.* **121**, 737–746 (2008). 18322270 10.1242/jcs.026096

[29] Gaiano, N. & Fishell, G. The role of notch in promoting glial and neural stem cell fates. *Annu. Rev. Neurosci.* **25**, 471–490 (2002). 12052917 10.1146/annurev.neuro.25.030702.130823

[30] Jalali, A. et al. HeyL promotes neuronal differentiation of neural progenitor cells. *J. Neurosci. Res.* **89**, 299–309 (2011). 21259317 10.1002/jnr.22562 PMC3079914

[31] Lowell, S. Stem cells: You make me feel so glial. *Curr. Biol.* **10**, R595–R597 (2000). 10985376 10.1016/s0960-9822(00)00636-9

[32] Meyers, E. A. & Kessler, J. A. TGF-beta family signaling in neural and neuronal differentiation, development, and function. *Cold Spring Harb. Perspect. Biol.* **9**, a022244 (2017). 10.1101/cshperspect.a022244 PMC5538418 28130363

[33] Russell, J. P. et al. Pituitary stem cells produce paracrine WNT signals to control the expansion of their descendant progenitor cells. *Elife* **10**, e59142 (2021). 10.7554/eLife.59142 PMC7803373 33399538

[34] Jin, S., Plikus, M. V. & Nie, Q. CellChat for systematic analysis of cell-cell communication from single-cell transcriptomics. *Nat. Protoc.* **20**, 180–219 (2025). 39289562 10.1038/s41596-024-01045-4

[35] Falix, F. A., Aronson, D. C., Lamers, W. H. & Gaemers, I. C. Possible roles of DLK1 in the Notch pathway during development and disease. *Biochim. Biophys. Acta* **1822**, 988–995 (2012). 22353464 10.1016/j.bbadis.2012.02.003

[36] Finn, J. et al. Dlk1-mediated temporal regulation of notch signaling is required for differentiation of alveolar type II to type I cells during repair. *Cell Rep.* **26**, 2942–2954 e2945 (2019). 30865885 10.1016/j.celrep.2019.02.046 PMC6464111

[37] Hamilton, A. K. et al. A proteogenomic surfaceome study identifies DLK1 as an immunotherapeutic target in neuroblastoma. *Cancer Cell* **42**, 1970–1982 e1977 (2024). 39454577 10.1016/j.ccell.2024.10.003 PMC11560519

[38] Crona, J. et al. Spatiotemporal Heterogeneity Characterizes the Genetic Landscape of Pheochromocytoma and Defines Early Events in Tumorigenesis. *Clin. Cancer Res.* **21**, 4451–4460 (2015). 25991818 10.1158/1078-0432.CCR-14-2854

[39] Papathomas, T. G. et al. SDHB/SDHA immunohistochemistry in pheochromocytomas and paragangliomas: a multicenter interobserver variation analysis using virtual microscopy: a multinational study of the European Network for the Study of Adrenal Tumors (ENS@T). *Mod. Pathol.* **28**, 807–821 (2015). 25720320 10.1038/modpathol.2015.41

[40] Parkinson, L. M. et al. The proneural transcription factor ASCL1 regulates cell proliferation and primes for differentiation in neuroblastoma. *Front. Cell Dev. Biol.* **10**, 942579 (2022). 36263020 10.3389/fcell.2022.942579 PMC9574099

[41] Guillemot, F. et al. Mammalian achaete-scute homolog 1 is required for the early development of olfactory and autonomic neurons. *Cell* **75**, 463–476 (1993). 8221886 10.1016/0092-8674(93)90381-y

[42] Nieto, M., Schuurmans, C., Britz, O. & Guillemot, F. Neural bHLH genes control the neuronal versus glial fate decision in cortical progenitors. *Neuron* **29**, 401–413 (2001). 11239431 10.1016/s0896-6273(01)00214-8

[43] Zethoven, M. et al. Single-nuclei and bulk-tissue gene-expression analysis of pheochromocytoma and paraganglioma links disease subtypes with tumor microenvironment. *Nat. Commun.* **13**, 6262 (2022). 36271074 10.1038/s41467-022-34011-3 PMC9587261

[44] Olsen, T. K. et al. Joint single-cell genetic and transcriptomic analysis reveal pre-malignant SCP-like subclones in human neuroblastoma. *Mol. Cancer* **23**, 180 (2024). 39217332 10.1186/s12943-024-02091-y PMC11365129

[45] Lo, L., Tiveron, M. C. & Anderson, D. J. MASH1 activates expression of the paired homeodomain transcription factor Phox2a, and couples pan-neuronal and subtype-specific components of autonomic neuronal identity. *Development* **125**, 609–620 (1998). 9435282 10.1242/dev.125.4.609

[46] Oudijk, L. et al. Immunohistochemical expression of stem cell markers in pheochromocytomas/paragangliomas is associated with SDHx mutations. *Eur. J. Endocrinol.* **173**, 43–52 (2015). 25916394 10.1530/EJE-14-1164

[47] Lee, S. et al. Neuronal apoptosis linked to EglN3 prolyl hydroxylase and familial pheochromocytoma genes: developmental culling and cancer. *Cancer Cell* **8**, 155–167 (2005). 16098468 10.1016/j.ccr.2005.06.015

[48] He, F. & Soriano, P. Sox10ER(T2) CreER(T2) mice enable tracing of distinct neural crest cell populations. *Dev. Dyn.* **244**, 1394–1403 (2015). 26250625 10.1002/dvdy.24320 PMC4619116

[49] Welander, J. et al. Rare germline mutations identified by targeted next-generation sequencing of susceptibility genes in pheochromocytoma and paraganglioma. *J. Clin. Endocrinol. Metab.* **99**, E1352–E1360 (2014). 24694336 10.1210/jc.2013-4375 PMC5393486

[50] Kowalczyk, M. S. et al. Single-cell RNA-seq reveals changes in cell cycle and differentiation programs upon aging of hematopoietic stem cells. *Genome Res.* **25**, 1860–1872 (2015). 26430063 10.1101/gr.192237.115 PMC4665007

[51] Satija, R., Farrell, J. A., Gennert, D., Schier, A. F. & Regev, A. Spatial reconstruction of single-cell gene expression data. *Nat. Biotechnol.* **33**, 495–502 (2015). 25867923 10.1038/nbt.3192 PMC4430369

[52] Yu, G., Wang, L. G., Han, Y. & He, Q. Y. clusterProfiler: an R package for comparing biological themes among gene clusters. *OMICS* **16**, 284–287 (2012). 22455463 10.1089/omi.2011.0118 PMC3339379

[53] Jin, S. et al. Inference and analysis of cell-cell communication using CellChat. *Nat. Commun.* **12**, 1088 (2021). 33597522 10.1038/s41467-021-21246-9 PMC7889871

[54] Li, B. & Dewey, C. N. RSEM: accurate transcript quantification from RNA-Seq data with or without a reference genome. *BMC Bioinformatics* **12**, 323 (2011). 21816040 10.1186/1471-2105-12-323 PMC3163565

[55] Puram, S. V. et al. Single-cell transcriptomic analysis of primary and metastatic tumor ecosystems in head and neck cancer. *Cell* **171**, 1611–1624 e1624 (2017). 29198524 10.1016/j.cell.2017.10.044 PMC5878932

[56] Puram, S. V. et al. Cellular states are coupled to genomic and viral heterogeneity in HPV-related oropharyngeal carcinoma. *Nat. Genet.* **55**, 640–650 (2023). 37012457 10.1038/s41588-023-01357-3 PMC10191634

