# Supplementary material for: Postnatal sustentacular cells as chromaffin progenitors and tumor cells of origin in VHL-related paragangliomas
Source: NPJ Precis Oncol. 2025 Oct 15;9:324. doi: 10.1038/s41698-025-01145-8 (PMC12528426; doi:10.1038/s41698-025-01145-8)
Supplement: Supplementary file 1 — Supplementary Information [file 41698_2025_1145_MOESM1_ESM.pdf]

Supplementary Materials:

Postnatal Sustentacular Cells as Chromaffin Progenitors and Tumor Cells of Origin in VHL-Related Paragangliomas

Petra Bullova† [1], Peng Cui† [1], Maria Arceo[1], Jiacheng Zhu[1], Wenyu Li[1], Valentin P[1], Monika Plescher[1], Katerina Stripling[1, 2], Christian Santangeli[1], Lidiya Mykhaylechko[1], Maria Eleni Kastriti[3], Catharina Larsson[1], C. Christofer Juhlin[1], Michael Mints[1*] and Susanne Schlisio[1*]

*Corresponding authors: Michael Mints michael.mints@ki.se, and Susanne Schlisio susanne.schlisio@ki.se.

This PDF file includes:

Supplementary Figures and legends S1 - S5
Supplementary Table legends S1 - S7

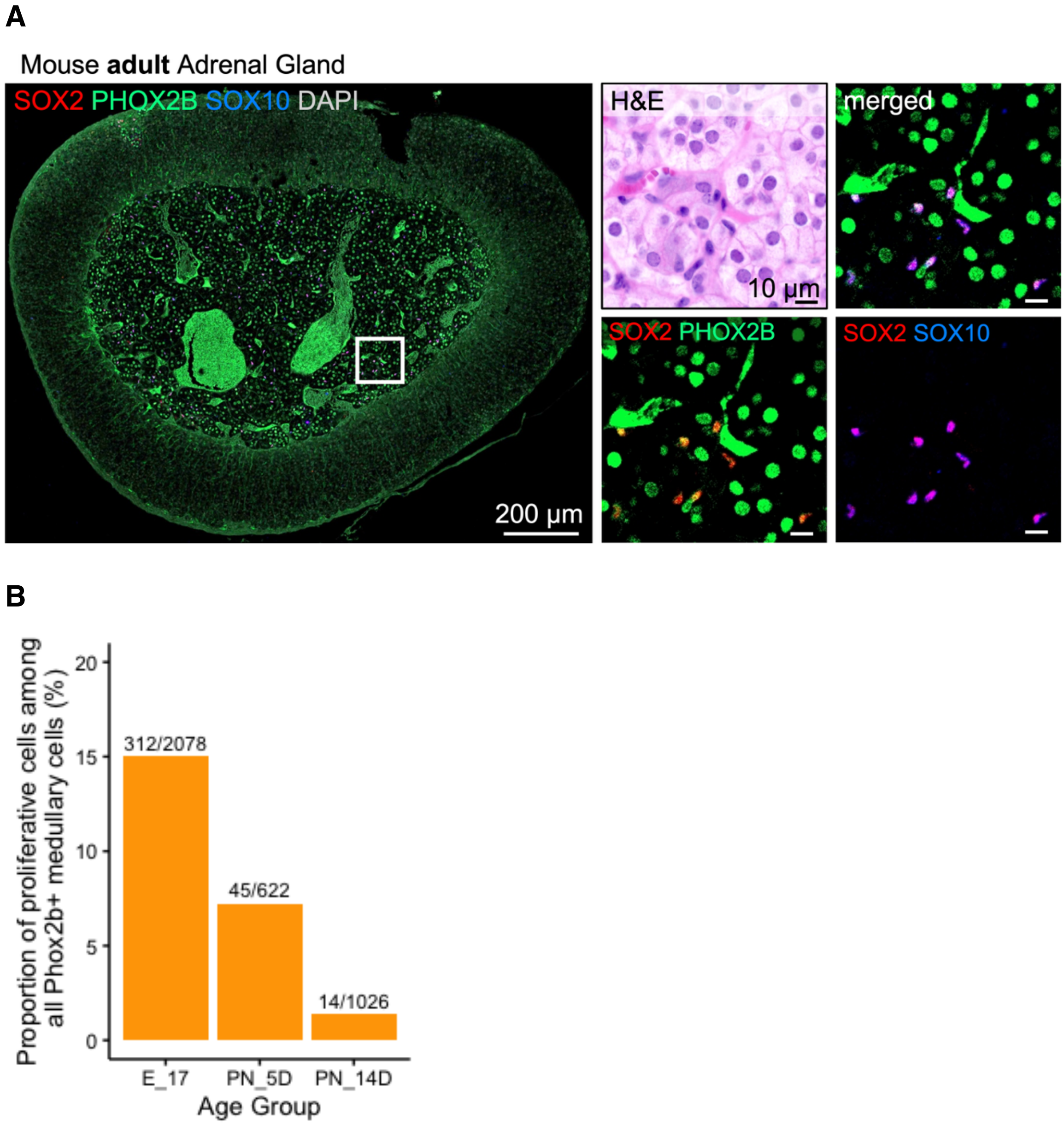

Suppl. Fig. S1

(A) Immunofluorescence and H&E staining of mouse adult adrenal gland showing adrenal medullary glial cells co-expressing SOX2 and SOX10. Scale bars in the adrenal glands and insets are 200µm and 10µm, respectively. **(B)** Quantification of proliferative PHOX2B^{+} medullary cells (MKI67^{+}, cluster 8) identified by single-cell RNA sequencing (see Figure 5) at embryonic day 17 (E17), postnatal day 5 (PN5), and postnatal day 14 (PN14). The bar graph shows the percentage of proliferative (cluster 8) cells relative to all PHOX2B^{+} medullary cells at each developmental stage.

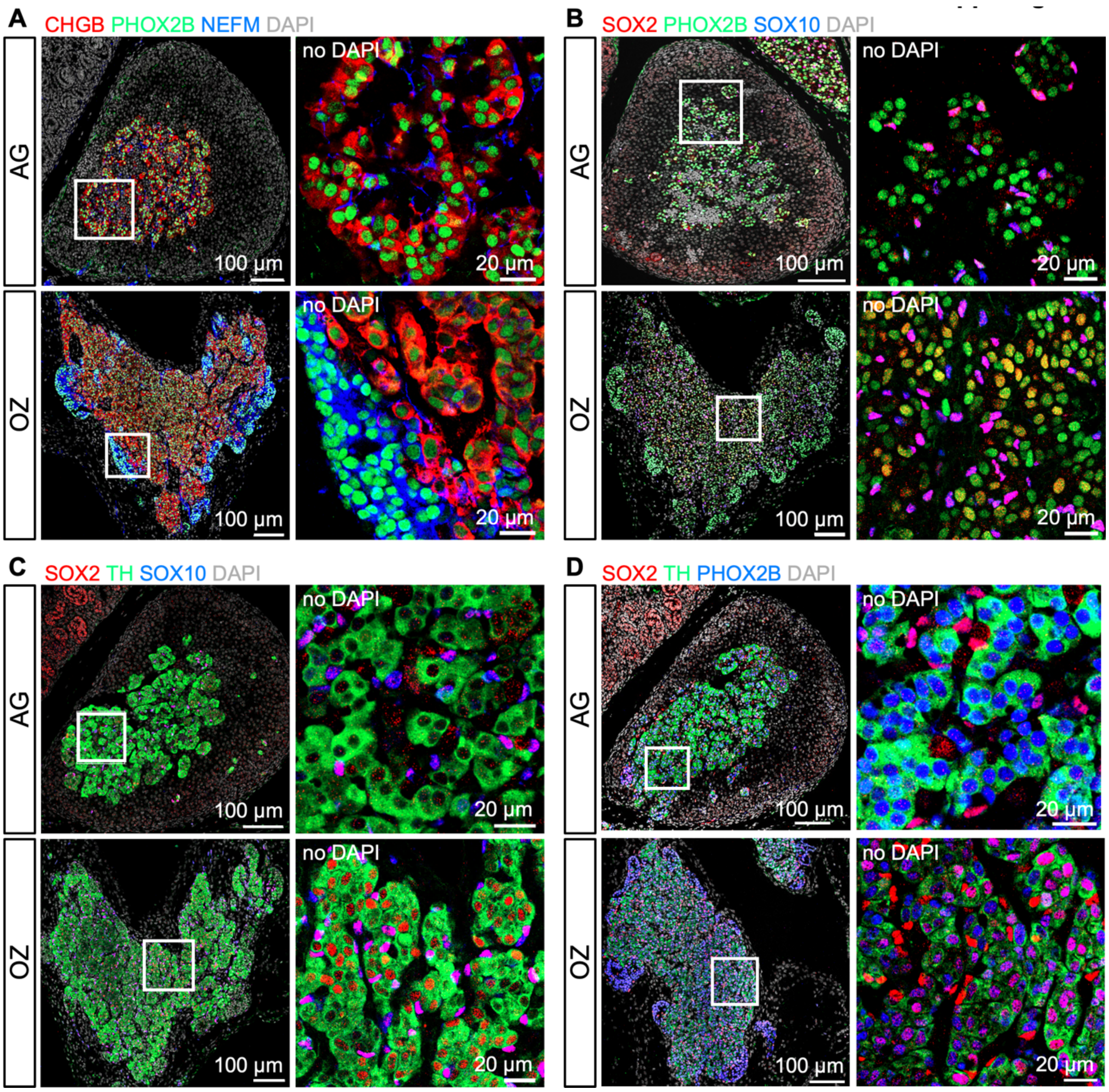

Suppl. Fig. S2

(A) Representative image showing immunofluorescence staining for CHGB, PHOX2B and NEFM in adrenal gland and organ of Zuckerkandl of P1 mice. **(B)** Representative image showing immunofluorescence staining for SOX2, PHOX2B and SOX10 in adrenal gland and OZ of P1 mice. **(C)** Representative image showing immunofluorescence staining for SOX2, TH and SOX10 in adrenal gland and organ of Zuckerkandl of P1 mice. **(D)** Representative image showing immunofluorescence staining for SOX2, TH and PHOX2B in adrenal gland and OZ of P1 mice. Scale bars in all adrenal glands or OZ images and insets are 100 µm and 20 µm, respectively.

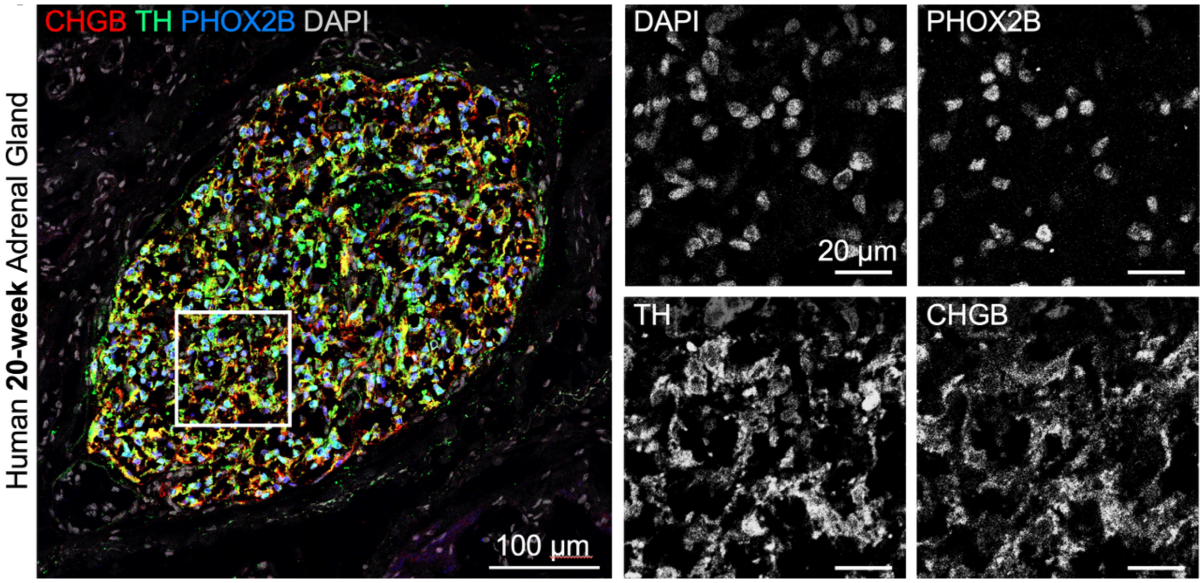

Suppl. Fig. S3A

(A) Representative image showing immunofluorescence staining for CHGB, TH and PHOX2B in 20-week-old human adrenal gland. Scale bars in the adrenal gland image and insets are 100 µm and 20 µm, respectively.

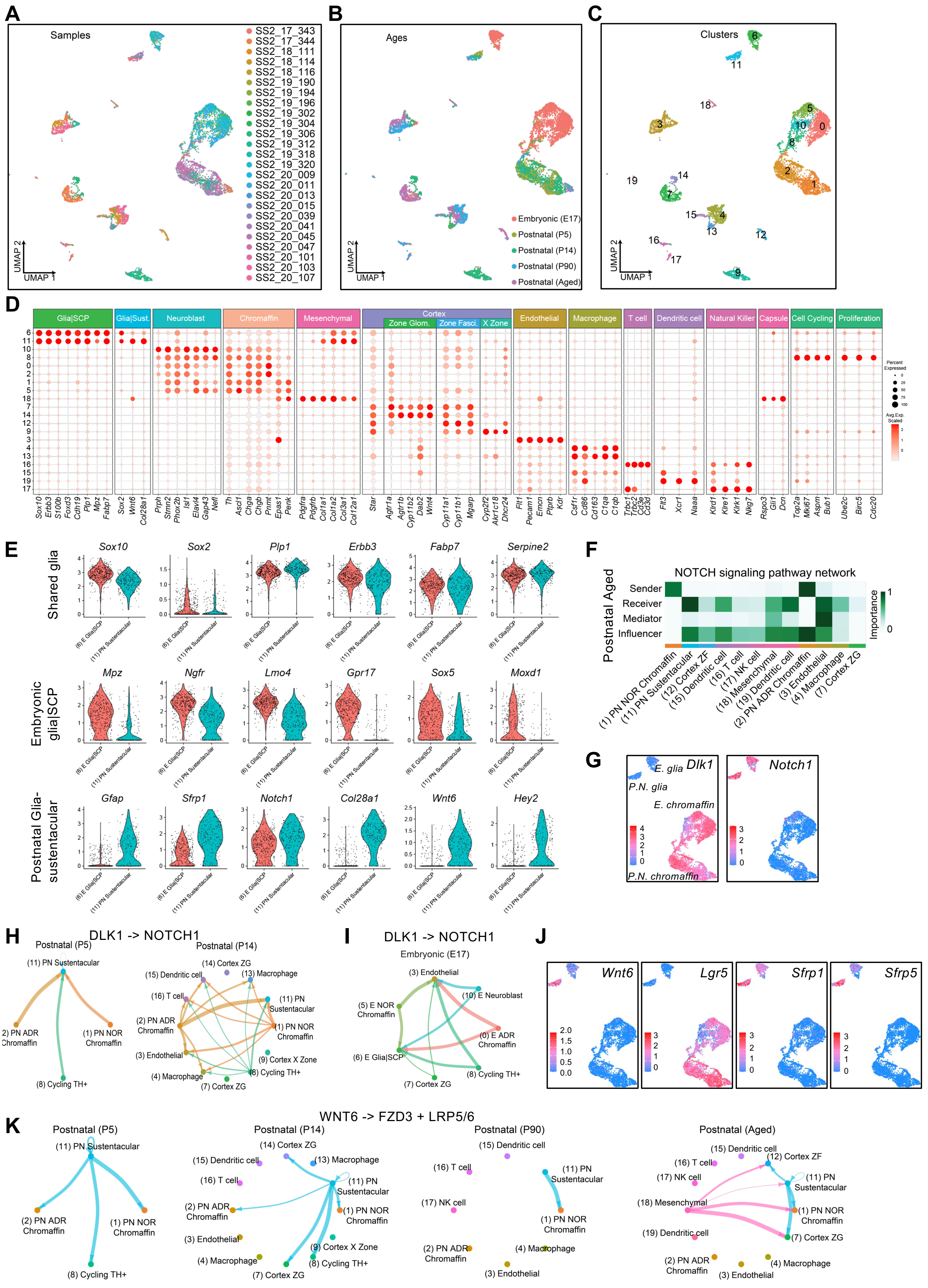

Suppl. Fig. S4

(A) UMAP plot showing sample identities of scRNA-Seq data of mouse adrenal tissue. **(B)** UMAP plot illustrating the detailed age grouping of cells from the mouse adrenal gland. **(C)** UMAP plot displaying cellular clusters identified in the scRNA-Seq data of mouse adrenal tissue. **(D)** Dot plot showing the expression patterns of cell type-specific marker genes across identified clusters in the scRNA-Seq dataset. **(E)** Violin plots display the expression levels of selected genes across two cell populations: "(6) E Glia|SCP" (red) and "(11) PN Sustentacular" (cyan). Genes are grouped into three categories: shared glial markers (top panel), embryonic glia-specific genes (middle panel), and postnatal glia-specific genes (bottom panel). **(F)** Heatmaps representing the relative centrality of each aged mouse adrenal gland cell group within the NOTCH signaling networks, assessed via four network centrality measures (sender, receiver, mediator, influencer). **(G)** UMAP plot illustrating the expression of *Dlk1* and *NOTCH1*. **(H)** Network diagram showing inferred DLK1-NOTCH1 ligand-receptor interactions among cell types in P5 and P14 mouse cells. **(I)** Network diagram showing inferred DLK1-NOTCH1 ligand-receptor interactions among cell types in E17 mouse cells. **(J)** UMAP plot illustrating the expression of Wnt6, Lgr5, Sfrp1, and Sfrp5. **(K)** Network diagram showing inferred WNT6 - (FZD3 + LPR5/6) ligand-receptor interactions among cell types in postnatal mouse cells.

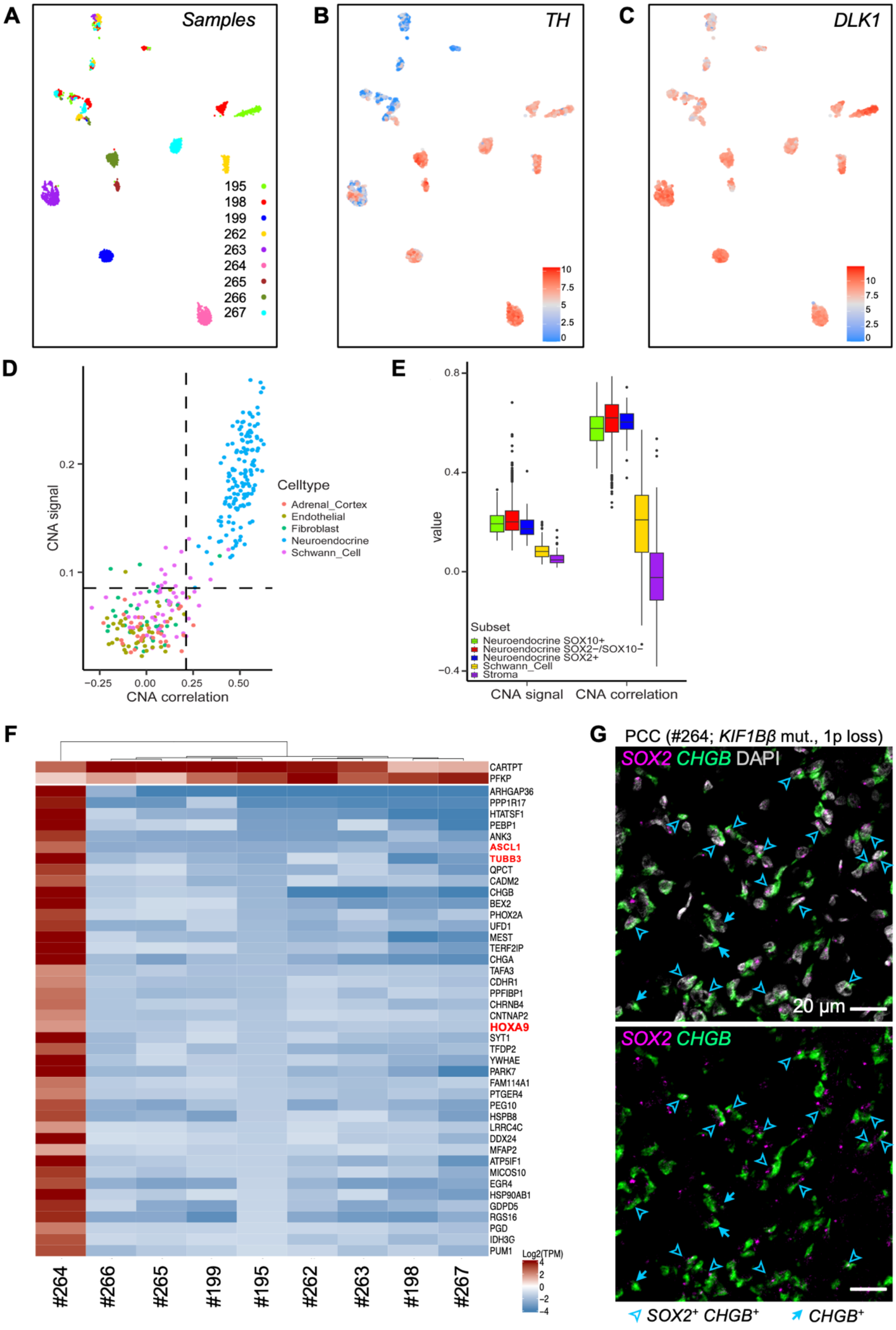
A
Samples
195
198
199
262
263
264
265
266
267
B
TH
C
DLK1
10
7.5
5
2.5
0
D
CNA signal
CNA correlation
Celltype
Adrenal_Cortex
Endothelial
Fibroblast
Neuroendocrine
Schwann_Cell
E
value
Subset
Neuroendocrine SOX10+
Neuroendocrine SOX2−/SOX10−
Neuroendocrine SOX2+
Schwann_Cell
Stroma
CNA signal
CNA correlation
F
CARTPT
PFKP
ARHGAP36
PPP1R17
HTATSF1
PEBP1
ANK3
ASCL1
TUBB3
QPCT
CADM2
CHGB
BEX2
PHOX2A
UFD1
MEST
TERF2IP
CHGA
TAFA3
CDHR1
PPFIBP1
CHRNB4
CNTNAP2
HOXA9
SYT1
TFDP2
YWHAE
PARK7
FAM114A1
PTGER4
PEG10
HSPB8
LRRC4C
DDX24
MFAP2
ATP5IF1
MICOS10
EGR4
HSP90AB1
GDPD5
RGS16
PGD
IDH3G
PUM1
Log2(TPM)
#264
#266
#265
#199
#195
#262
#263
#198
#267
G
PCC (#264; KIF1Bβ mut., 1p loss)
SOX2 CHGB DAPI
20 µm
SOX2 CHGB
SOX2+ CHGB+
CHGB+

Suppl. Fig. S5

(A) UMAP of 2,586 single nuclei colored by sample of origin. **(B)** UMAP plot illustrating the expression of *TH*. **(C)** UMAP plot illustrating the expression of *DLK1*. **(D)** Malignant cell definition: Example from sample #198. The y-axis represents CNA signal and the x-axis CNA correlation (see Methods). Each point is a cell, colored by assigned cell type. Lines represent cutoffs (median + 2SD of adrenocortical, fibroblast and endothelial cells). Only cells passing both cutoffs were defined as malignant. **(E)** CNA metrics for $SOX2^{+}SOX10^{+}$ neoplastic cells. Boxplots showing CNA signal and CNA correlation for neuroendocrine cells, split by SOX2/SOX10 expression, Schwann cells and stroma (all fibroblasts, endothelial and adrenocortical cells). **(F)** Recurrently differentially expressed genes in PPGL #264**.** Rows are genes significantly over/under expressed in all paired comparisons between malignant cells from sample #264 and each other tumor sample. Columns are mean expression values of each gene per sample. All values were centered by the mean expression in sample #264. **(G)** *SOX2* and *CHGB* RNAscope *in situ* hybridization in PPGL #264. Empty arrowheads indicate $SOX2^{+}CHGB^{+}$ double-positive cells and full arrows indicate $CHGB^{+}$ cells. Scale bars are 20 µm.

Supplementary Table Legends:

Suppl. Table S1. Specific up-regulated differentially expressed gene list from scRNA-Seq data of mouse adrenal gland clusters defined in Fig. 5A (*P.adj* < 0.05). *P* values are adjusted using Bonferroni correction (as detailed in Methods).

Suppl. Table S2. Mouse postnatal glia enriched gene ontology (GO) terms shown in Fig. 5F (*P.adj* < 0.05). Postnatal glia specific differentially expressed gene (as defined in Suppl. Table S1) were used as input for GO analysis. *P* values are adjusted using Benjamini-and-Hochberg (BH) methods (as detailed in Methods).

Suppl. Table S3. List of PPGL samples and corresponding mutation status from Fig. 6 and S5. MUT = Mutant. WT = Wild type. Const = constitutive.

Suppl. Table S4. Genes significantly up- or downregulated in all pairwise comparisons of PPGL sample #264 versus every other sample.

Suppl. Table S5. Differentially expressed genes between malignant and nonmalignant glia (referred as Schwann cell like cells, SCLCs) as previously defined in Zethoven et al. dataset [1].

Suppl. Table S6. Top 50 differentially expressed genes per cluster across all cells defined in Fig. 6A.

Suppl. Table S7. Top 50 differentially expressed genes per cluster in the tumor microenvironment (TME).
